# Evidence for the Robustness of Protein Complexes to Inter-Species Hybridization

**DOI:** 10.1371/journal.pgen.1003161

**Published:** 2012-12-27

**Authors:** Jean-Baptiste Leducq, Guillaume Charron, Guillaume Diss, Isabelle Gagnon-Arsenault, Alexandre K. Dubé, Christian R. Landry

**Affiliations:** Institut de Biologie Intégrative et des Systèmes, Département de Biologie, PROTEO, Pavillon Charles-Eugène-Marchand, Université Laval, Québec City, Canada; University of Michigan, United States of America

## Abstract

Despite the tremendous efforts devoted to the identification of genetic incompatibilities underlying hybrid sterility and inviability, little is known about the effect of inter-species hybridization at the protein interactome level. Here, we develop a screening platform for the comparison of protein–protein interactions (PPIs) among closely related species and their hybrids. We examine *in vivo* the architecture of protein complexes in two yeast species (*Saccharomyces cerevisiae* and *Saccharomyces kudriavzevii*) that diverged 5–20 million years ago and in their F1 hybrids. We focus on 24 proteins of two large complexes: the RNA polymerase II and the nuclear pore complex (NPC), which show contrasting patterns of molecular evolution. We found that, with the exception of one PPI in the NPC sub-complex, PPIs were highly conserved between species, regardless of protein divergence. Unexpectedly, we found that the architecture of the complexes in F1 hybrids could not be distinguished from that of the parental species. Our results suggest that the conservation of PPIs in hybrids likely results from the slow evolution taking place on the very few protein residues involved in the interaction or that protein complexes are inherently robust and may accommodate protein divergence up to the level that is observed among closely related species.

## Author Summary

Independently evolving lineages accumulate mutations that are compatible within lineage but that may be incompatible among lineages. These incompatibilities are expected to accumulate among gene products that act together to produce a phenotype or that interact with each other physically. Genes coding for proteins that assemble into protein complexes co-evolve with each other in order to maintain these interactions over evolutionary time. These protein complexes are therefore potential hotspots for the accumulation of molecular incompatibilities. Because of the lack of molecular tools, this question has not been systematically addressed. Here, we develop a platform to measure, *in vivo*, the divergence of protein complexes in different yeast species and their perturbation in their F1 hybrids. We find that, despite a level of protein divergence that is as high as that observed between birds and mammals, most protein–protein interactions are highly conserved between species and are not perturbed in hybrids. Contrary to our expectations, our results show that protein complexes may be robust to inter-species hybridization and may not be a major contributor of incompatibilities to the reproductive isolation of recently formed species.

## Introduction

Identifying the molecular processes underlying species incompatibilities is a fundamental issue in evolutionary biology. Charles Darwin first suggested that the inviability and sterility often occurring after species hybridization could result from mechanisms other than the differences observed between parents and morphological disorders in the descendants [1]. Several theoretical models have since been developed to address how differences leading to incompatibilities may accumulate between species [2]–[6] and genetic mapping of such incompatibilities [7]–[10] has uncovered some molecular mechanisms by which they may take place. In theory, any system in which molecules or genes interact with each other to perform a function and that co-evolve within species, could give rise to such incompatibilities. Most studies on molecular incompatibilities have so far been performed using genetic approaches to identify the genes involved in hybrid lethality and sterility, for instance, in *Drosophila* [7]–[9] or in hybrid sterility in yeasts [10]. In a cell, all processes are controlled and mediated by protein-protein interactions (PPIs). Stable PPIs form protein complexes that are the workhorses of the cell and perform fundamental tasks such as DNA replication, repair and transcription, cellular transport or cytoskeleton architecture [11]–[16]. More transient interactions are involved, for instance, in cell signaling events such as phosphorylation cascades [17]. Proteins are suggested to co-evolve within species over-time as a response of selection to maintain these interactions [18]–[20], and this co-evolution is so important that it can be used to infer PPIs [20]. Each pairwise PPI therefore represents an opportunity for incompatibilities to accumulate between closely related species, although there is limited empirical evidence for this phenomenon [21]. Whether protein divergence can result in incompatibilities in protein complexes in hybrids is largely unknown because we lack experimental systems to directly measure physical interactions in living cells in closely related species and in inter-species hybrids.

Here we develop such a system using the budding yeast *Saccharomyces cerevisiae* (*Scer*) and its closely related species belonging to the *Saccharomyces sensu stricto* group [22], [23]. As its interactome has been intensely studied, we reasoned that the budding yeast would be an appropriate reference for protein interaction network (PIN) comparisons among species and in hybrids between species. More than 70,000 physical interactions have been reported in major protein interaction databases such as the BioGRID [24] for this species. Another reason why *Scer* is a particularly good model to study the implication of genetic incompatibilities in hybrids is the availability of closely related species that are genetically tractable [23] and that can be mated with each other. Indeed, F1 hybrids among *Saccharomyces* species are viable and are found in the wild [25] and can be readily produced in the laboratory [26]. However, they show strong incompatibilities, as they are almost entirely sterile [27], [28], which makes standard genetic mapping approaches inefficient to identify incompatibilities systematically.

Several techniques are available for interactome mapping and could be applied to the screening of abnormal PPIs directly in viable F1 hybrids. A technique that would be particularly powerful would integrate all factors that affect PPIs such as protein abundance and localization. It would allow detecting PPIs *in vivo*, among proteins expressed at their endogenous levels and at their normal cellular localization. One such method has recently been developed for the budding yeast and is based on the Protein-fragment Complementation Assay (PCA) technology [13], [29]. This technique detects structural relationships among proteins, as the C-termini of both proteins need to be within 80 Angstroms of each other for PPI detection [13]. The signal detected also reflects the amount of protein complexes formed [30]. Here, we set out to adapt the PCA based on the dihydrofolate reductase (DHFR), recently developed for *Scer* [13], to other species of the *Saccharomyces sensu stricto* group, which covers the same range of molecular divergence as that between birds and mammals [22]. Using this tool, we directly test whether 5–20 millions years of evolution result in protein incompatibility between species, *i.e.* in gain or loss of PPIs in the hybrid background as compared to the parental species.

**Figure 1:**
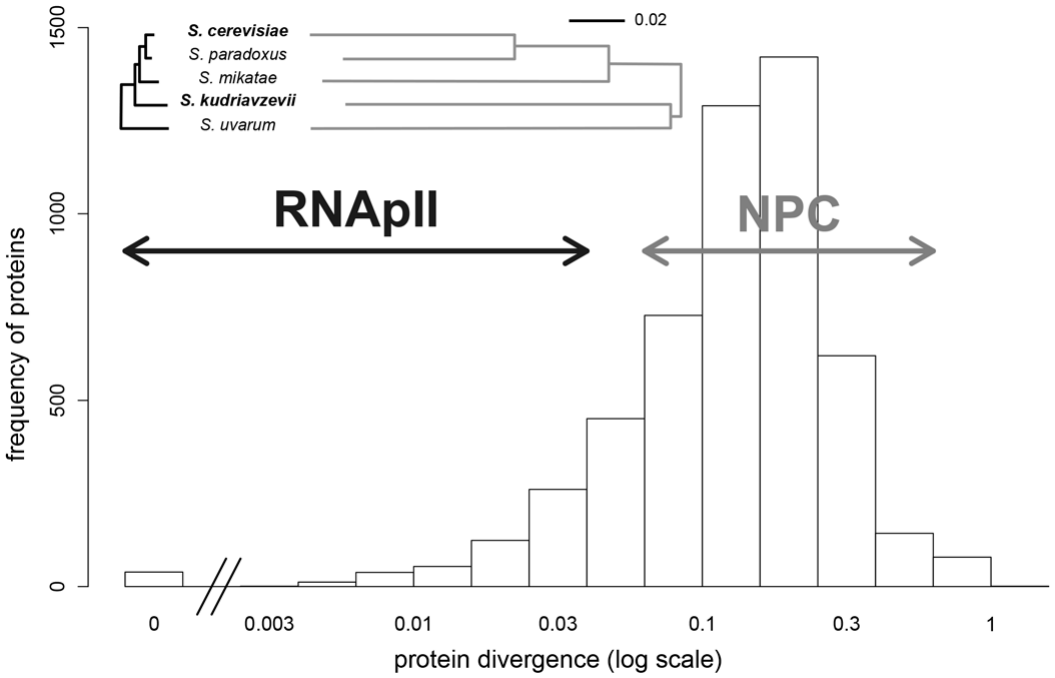
Protein divergence in the RNApII (black) and the NPC (grey) in the *Saccharomyces sensu stricto* group. Evolutionary trees were drawn for all proteins of a complex and are on the same scale (proportion of different amino acids). Distribution of protein divergence between *Scer* and *Skud* was calculated from multiple-sequence alignments available for 5261 orthologous proteins [23]. Arrows indicate protein divergence covered by the two complexes.

We identified two protein complexes that can serve as models, namely the nuclear pore complex (NPC) and the RNA polymerase II (RNApII). We selected these two complexes because they have distinct functions and contrasting patterns of evolution. The NPC performs selective molecule transport across the nuclear envelope and its overall structure is conserved among eukaryotes [31], although the precise organization diverges among distantly related species [32]. The RNApII is a core enzyme that performs DNA transcription and its structure is fully conserved in eukaryotes [33]. The structure of these complexes has either been well established or extensively modeled. In *Scer*, the NPC is a circular assembly of about 500 proteins (nucleoporins) arranged in eight identical substructures, each constituted of about 30 nucleoporins, most of them being duplicated in a substructure [34]. The RNApII is composed of 12 interacting subunits [35]. These complexes were at least partially detected *in vivo* in *Scer* by DHFR-PCA [13] and in agreement with structural information provided by other techniques [24]. A number of characteristics make these complexes appropriate models to investigate the impact of hybridization on PPIs. First, proteins belonging to the same complex evolve at equivalent rates in *Saccharomyces* species (Figure 1), reflecting the potential coevolution of subunits within NPC and RNApII, as expected from previous reports [36]. Further, the NPC proteins are at the high end of the spectrum of divergence among the *Saccharomyces* species, whereas RNApII proteins show stronger conservation (Figure 1). Second, the NPC and RNApII represent contrasted situations because their respective functions require different levels of complex architecture flexibility. For instance, in *Scer*, most of RNApII subunits are essential for cell viability [37] and all are required for transcription [35], whereas about one third of nucleoporin genes are non-essential [37] and many NPC proteins perform redundant structural functions and are thus likely interchangeable [34]. Also, the *Scer* NPC includes several recent genes which originated during the whole yeast genome duplication [38]. All these observations suggest that PPIs in the NPC would be more likely to diverge among species. A lack of changes in the architecture of the NPC between species or in the hybrid would suggest that protein complexes can be robust to protein divergence.

## Results

### The DHFR-PCA permits detection of PPIs across the *Saccharomyces sensu stricto* species complexes

**Figure 2:**
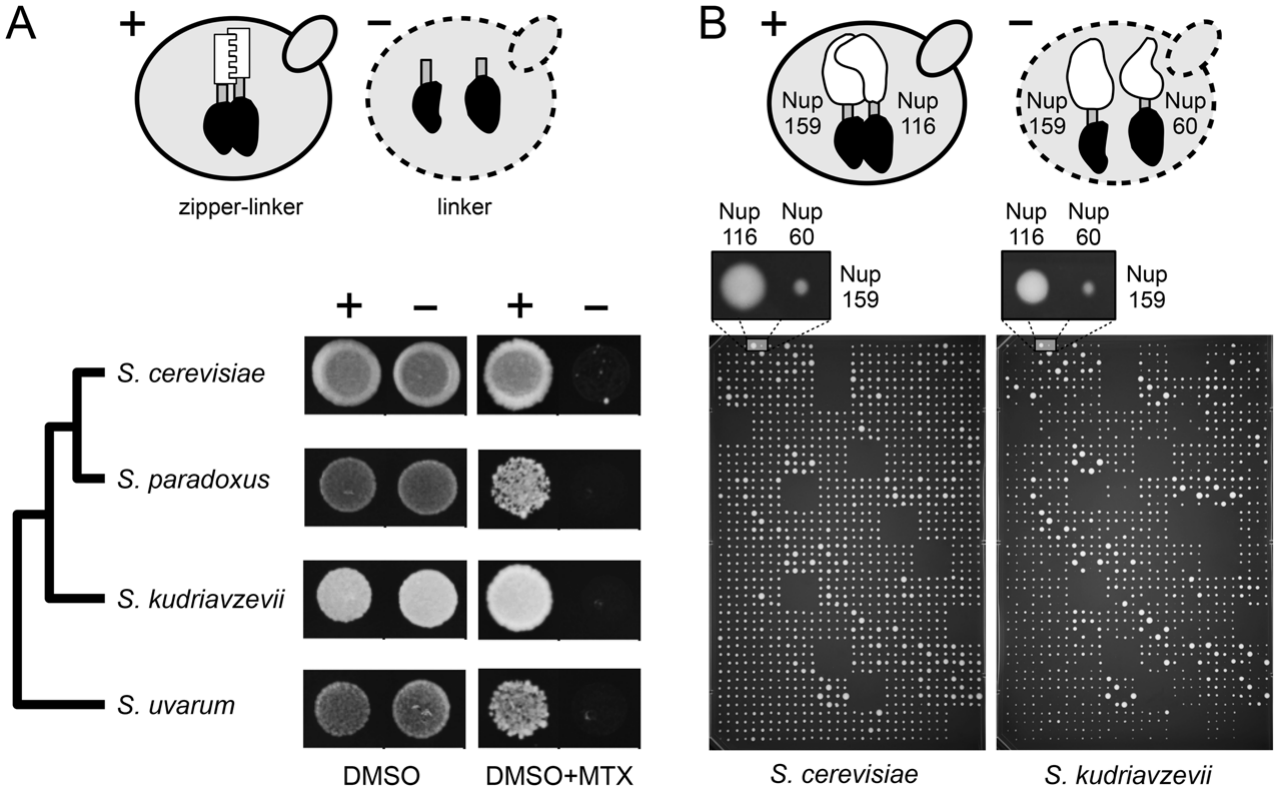
Optimization of the DHFR-PCA in four *Saccharomyces* species. (*A*) Control diploid strains were grown in presence of DMSO (control) and methotrexate (MTX). In the positive controls (+), two zipper protein fragments (white) were fused via a linker (grey) to complementary protein fragments of the MTX-resistant DHFR (black). Zipper fragments strongly interact, reconstituting the DHFR activity and allowing growth on MTX medium. In the negative controls (−), the absence of interaction (L alone) prevents the DHFR reconstitution and strain growth. Optimal signal was found in *Scer* and *Skud*. (*B*) High density arrays were used to screen PPIs in *Scer* and *Skud*. Each plate contained crosses between 24 *MATa* and 16 *MATα* strains in three non adjacent replicates. Here, the interaction between Nup159 and Nup116 reconstitutes the DHFR (+) and allows growth of the colony in both species. Nup159 and Nup60 do not interact directly (−) and strains do not grow on MTX medium.

We first tested whether we could detect PPIs using the DHFR-PCA in *S. paradoxus* (*Spar*), *S. uvarum* (*Suva*) and *S. kudriavzevii* (*Skud*) and determined what would be the optimal experimental conditions for measuring PPIs *in vivo*. The DHFR-PCA assay is based on the reconstitution of the DHFR enzyme (see Figure S1 *A*–S1*B*). In order to measure the signal to noise ratio of this assay in the four species, we engineered a strong PPI reporter by fusing the DHFR fragments (F[1], [2] and F[3]) downstream of the coding sequence of homodimerizing residues of the GCN4 parallel coiled-coil leucine zipper [39]. Overall, we observed the strongest signal-to-noise ratio (growth of positive/negative controls) in *Scer* and *Skud* and in conditions that are standard for the *Scer* DHFR-PCA (Figure 2*A*). We thereafter focused our study on *Scer* and *Skud* to ensure optimal conditions to detect PPIs in these species and in their hybrids. The two species show an average of 16.4% pairwise protein divergence (Figure 1), which is in the same order of magnitude than what it is observed between birds and mammals [22]. Because no hybridizable species presents such a protein divergence level in plants or animals, *Scer* and *Skud* offer a conservative system to measure how hybrid protein complexes form in inter-species crosses.

### Comparison of protein complexes between species and their hybrids

We set out to compare the architecture of the NPC (15 subunits considered) and the RNApII (9 subunits considered) between *Scer*, *Skud* and their F1 hybrids. The 24 subunits corresponded to a subset of *Scer* proteins, which were showed to be involved in at least one PPI in the considered complexes in Tarrassov *et al.* [13]. Not all *Scer* strains were available in the DHFR-PCA collection so we reconstructed them by homologous recombination as described in Tarrassov *et al.* [13]. We then attempted to construct all of the equivalent strains (24 in *MATa* and 24 in *MATα*) in *Skud* by targeting the orthologous genes [40]. All strains were obtained for *Scer*; 23 and 17 strains were obtained for, respectively, *MATa* and *MATα* strains in *Skud* (Table S1). We then performed a comprehensive screen of PPIs within and between the NPC and RNApII, within species and their hybrids. We crossed all *Scer* and *Skud MATa* strains with all *Scer* and *Skud MATα* strains. Most of interactions between two proteins P1 and P2 were testable in two reciprocal ways (P1 in *MATa*×P2 in *MATα vs.* P2 in *MATa*×P1 in *MATα*; Figure S2 *A*), which was the case of 276 PPIs. The 24 remaining homomeric interactions (P1 in *MATa*×P1 in *MATα*) were also tested. We compared these PPIs between *Scer*, *Skud* and the inter-specific hybrids, namely hybrid 1 (*Scer MATa*×*Skud MATα*) and hybrid 2 (*Skud MATa*×*Scer MATα*), giving a total of 1927 possible combinations (Table S2). In order to evaluate the interaction between P1 from *Scer* and P2 from *Skud*, P1-P2 was measured in hybrid 1 and P2-P1 in hybrid 2 (Figure S2 *A*). All combinations P1-P2 and P2-P1 were replicated three times. PPIs were measured by estimating colony size in pixels on high quality images of plates (Figure 2*B*). The DHFR-PCA signal was highly reproducible and the reproducibility was similar for the intra and inter-specific crosses (r = 0.90 to 0.97, *p*<0.001; Figure S3). Triplicates were averaged, giving a mean growth signal index (SI) for all downstream analyses (data available in Dataset S1). Because SI values associated to a particular protein were sometimes lower when the protein was tagged in *MATa* or *MATα*, potentially generating false positive or hide true interactions, we corrected SI values for these haploid strain effects (see Methods and Figures S2B–S2F, S4, S5, S6, S7, data available in Dataset S2).

**Figure 3:**
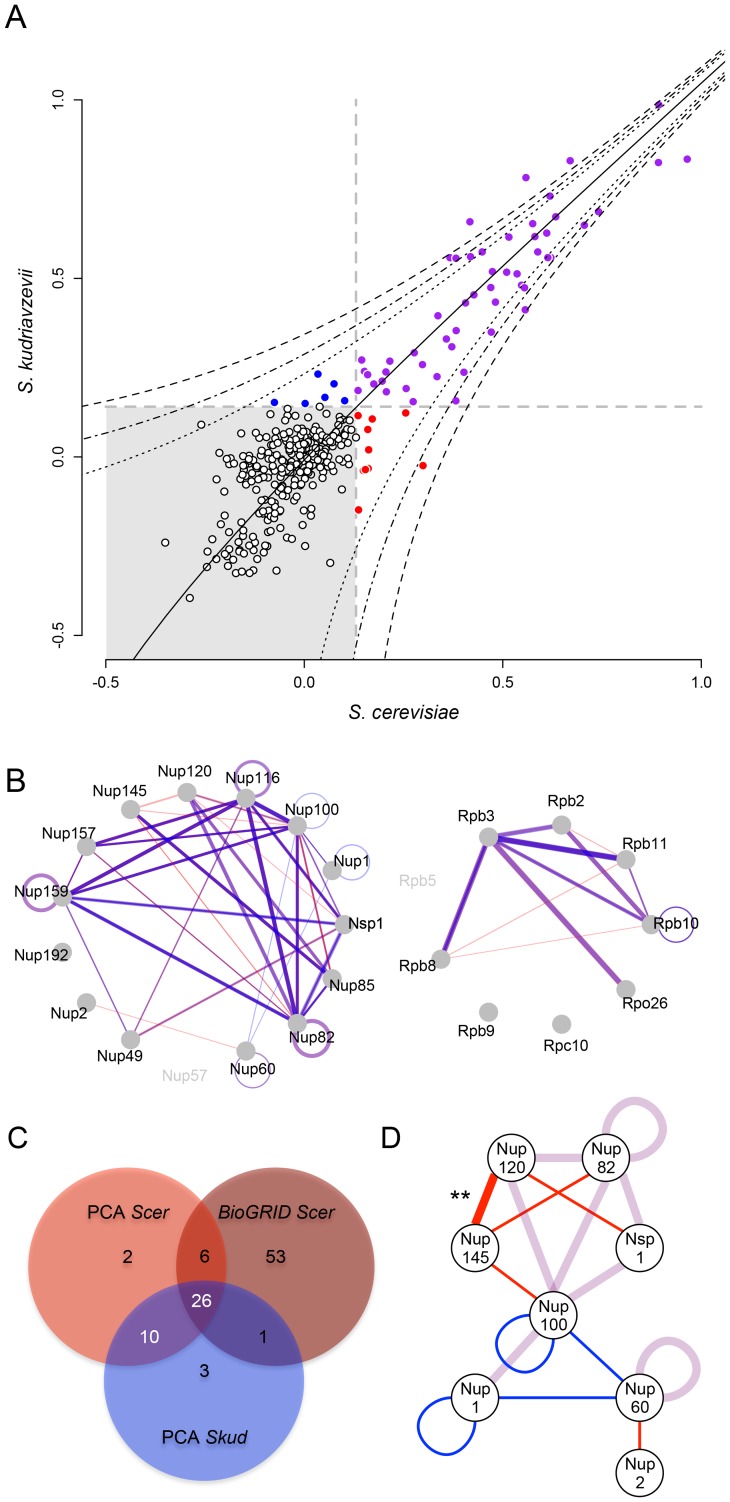
The NPC and RNApII networks are largely conserved between *Scer* and *Skud*. (*A*) Comparison of SI values (strains growth signal index; log_10_) between species. Grey dotted lines indicate SI threshold values (*t*). SI values were considered to correspond to interactions when greater than *t* in both species (purple) or specific to one species when greater than *t* only in *Scer* (red), or only in *Skud* (blue). Black dotted lines indicate 5%, 1% and 0.1% threshold above which SI residuals significantly deviate from the *Scer-Skud* regression (black line). (*B*) Overlapped networks of *Scer* and *Skud*. Only SI above *t* and comparable interactions are represented. Line width is proportional to SI values measured between proteins in *Scer* (red) and *Skud* (blue) in the NPC (left) and the RNApII (right). Interactions appear in purple when *Scer* and *Skud* SI values overlap. Different degrees of purple depend on whether the interaction could be tested in reciprocal ways or not. (*C*) Venn diagram indicating the overlap of PPIs detected by PCA of other methods (BioGRID) in *Scer*, and by PCA in *Skud*. Reciprocal combinations of PPIs were collapsed. (*D*) Representation of PPIs shared (purple lines) or unique to *Scer* (red lines) or *Skud* (blue lines) in the NPC. Only proteins involving divergences in PPIs are showed. Only the difference in the Nup120-Nup145 PPI was significant (**: *p*<0.01).

We defined a threshold (*t*) above which SI would represent a PPI by comparing the distributions of SI measured among (SA) and within (SW) the NPC and RNApII (Figure S8 and S9), assuming that among complex crosses represent background growth of the colonies, which is well supported by the almost complete absence of physical interactions reported between NPC and RNApII proteins [24]. As all SA values fell below *t*≈1.4, we considered SW values above 1.4 to be a conservative threshold (Figure S8 and S9). In *Scer*, 39 out of 54 interactions (corresponding to 82 SW values with SW>*t* before reciprocal redundant combinations were collapsed) were concordant with physical interactions already reported in this species by independent methods (Figure S10 *A*). Also, SW values measured in *Scer* were positively correlated to the number of times a PPI was reported in BioGRID (*r* = 0.55, *p*<0.001; Figure S10 *B*). In *Scer*, PPIs were mostly detected between proteins for which C-termini are close to each other in the case of RNApII (Figure S10 *C*) or corresponded to the architecture of NPC previously defined (Figure S10 *D*). We observed a strong and highly significant correlation between SI values measured in the two species (*r* = 0.93, *p*<0.001; Figure 3*A*), suggesting a high degree of conservation in the protein complexes. Most interactions were shared between species, and SI remained highly correlated between species when only considering these interactions (*r* = 0.87, *p*<0.001). Among the 54 PPIs observed in *Scer*, 44 were comparable with *Skud* and 36 physical interactions were common to both species (8 in the RNApII, 28 in the NPC, Figure 3*A*–3*C*), corresponding to 50 significant SI values before reciprocal redundant combinations were collapsed. The remaining unshared PPIs in *Scer* and *Skud* (eight and four PPIs respectively) were all close to the detection threshold *t* and further examination by regression analysis showed that most of the differences were not significant (Figure 3*D*). This was the case for three very weak PPI signals found in RNApII in *Scer* but not in *Skud* (Figure 3*B* and Figure S10 *C*). Other differences involved nucleoporins Nup1, Nup2 and Nup60 belonging to the subcomplex of the nuclear pore basket, with respectively one and three PPIs specific to *Scer* and *Skud* (Figure 3*D*). The structure of this subcomplex is poorly known [41] mainly because its location at the nuclear side makes unsuccessful the use of most of PPIs detections methods, which was in agreement with weak signals we found for these interactions. Thus, like the differences we found in RNApII, these variations have to be carefully interpreted. Other variations in PPIs involved proteins from the outer ring NPC subcomplex. Four PPIs were absent in *Skud* and three of them involved interactors of Nup145; Nup120, Nup82 and Nup100 (Figure 3*D*). However, the only statistically significant difference we found was the absence of Nup120-Nup145 interaction in *Skud*, which we discuss in-depth below (Figure 3*D*).

### Protein complex architecture in the hybrid background

**Figure 4:**
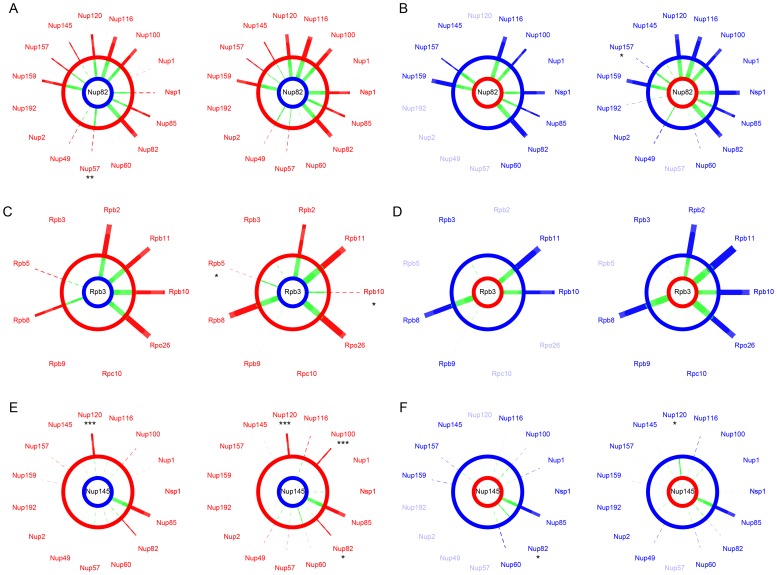
The NPC and RNApII are robust to hybridization. SI values compared between *Scer* (red) or *Skud* (blue) and *Scer-Skud* hybrids (green). Examples of proteins Nup82 (*A–B*) and Nup145 (*E–F*) in the NPC and Rpb3 (*C–D*) in the RNApII. Segment width is proportional to SI (dotted line if SI<*t*). For each comparison, networks on left show interactions measured between the protein of interest tagged in *MATa* (center) and other proteins of the same complex tagged in *MATα*. Networks on right show the reciprocal interactions. The protein of interest comes from *Scer* (red ring) or *Skud* (blue ring) and is in the species background (outer ring) or in the hybrid background (inner ring). Asterisks indicate whether the SI value measured in hybrid is significantly different from that measured in species (*: *p*<0.05; **: *p*<0.01; ***: *p*<0.001). Only the absence of *Scer*Nup120-*Skud*Nup145 is significant in reciprocal comparisons (*E*). Protein names were blurred when strains were unavailable.

We assessed the extent to which the integrity of the protein complexes was preserved in hybrids by comparing PPIs in their parental background to PPIs in F1 hybrids. In hybrids, protein complexes can be formed with subunits from the two species. Incompatibilities between subunits could directly disrupt interactions between the two proteins of interest or indirectly affect the architecture of the complex and disrupt or lead to novel, spurious, unexpected interactions [42]. We considered that an interaction was disrupted or gained in hybrids when its residual value resulting from the species/hybrid comparison was significantly different from the distribution of all residual values pooled together (Figure S2 *E*–S2*F*). Overall, we found that all interactions that were conserved between parental species were also seen in the hybrids, *i.e.* no interaction was disrupted specifically in the hybrid. We also found no instance of hybrid-specific interactions. For instance, we found that interactions involving Nup82 in the *Scer* background were fully conserved in hybrids between the *Skud*Nup82 and *Scer* NPC subunits (Figure 4*A*–4*B*). Similar observations were made for Rpb3 in RNApII (Figure 4*C*–4*D*). Additionally, SI values from species and hybrids were strongly correlated in most of cases (FigureS S4, S5, S6, S7), suggesting that inter-species PPIs could not be differentiated from within species PPIs. We observed the same pattern for almost all comparisons between hybrids and *Scer* (Figure S8) and *Skud* (Figure S9).

**Figure 5:**
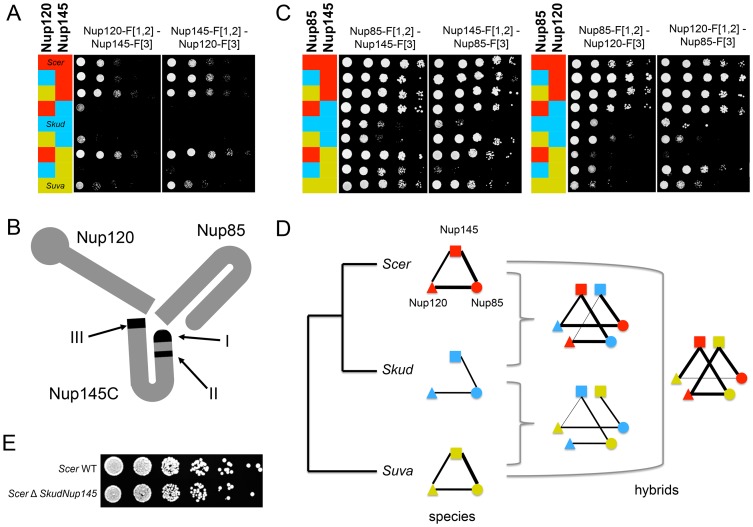
The absence of Nup120-Nup145C interaction in *Skud* is likely a PPI loss. (*A*) Spot assays on Methotrexate medium (six days of growth at 30°C) to dissect the interaction in *Scer* (red), *Skud* (blue), *Suva* (yellow) and their hybrids. The interaction between Nup145C from *Scer* and Nup120 from *Scer*, *Skud* or *Suva* is detected, whereas it is lost when Nup145C comes from *Skud*. The interaction was also absent when it involved Nup120 from *Skud* and Nup145 from *Suva*. (*B*) Schematic structure of the *Scer* Nup120-Nup85-Nup145 sub-complex adapted from Fernandez-Martinez *et al.* [43]. The three interacting domains are indicated in black. (*C*) Interactions between Nup85 and Nup145C or Nup120 in three species and their hybrids confirm that not all Nup145C interactions are lost. (*D*) Evolutionary tree of *Scer*, *Skud* and *Suva* and schematic representation of Nup145-Nup120-Nup85 interactions in species and hybrids according to spot assays (*A–C*), revealing several other loss of interaction in hybrids with *Suva*. Line width is proportional to the number of spot growth observed for each interaction. (*E*) Similar growth for BY4741 *Scer* wild type (WT) and modified strains (Δ*Skud-NUP145*) suggest that *Skud-Nup145* complements the absence of *Scer-Nup145* (YPD medium, two days of growth at 30°C), as *Nup145* is essential for growth in *Scer*.

We found one exception for the interaction between proteins Nup120 and Nup145, which are also involved in few divergent interactions between *Scer* and *Skud*. The Nup120-Nup145 interaction is present in *Scer* but is undetectable in *Skud* (Figure 3*D*), neither in hybrids between the *Scer*Nup120 and the *Skud*Nup145 (Figure 4*E*). Conversely, the *Skud*Nup120-*Scer*Nup145 interaction was observed (Figure 4*F*), suggesting that the absence of interaction in *Skud* is due to changes in the amino acid sequence of Nup145 rather than Nup120. In order to determine whether the Nup145-Nup120 interaction was lost in *Skud* or gained in *Scer*, we repeated the PCA experiment between Nup145 and Nup120 by including *Suva*, which diverged early in the *Saccharomyces sensu scricto* group. As shown in Figure 5*A*, we found that the Nup145-Nup120 interaction was present in *Suva*, suggesting that the absence of interaction in *Skud* is a lost rather than a gain in *Scer*. Figure 5*B* shows three protein domains of Nup145 (namely I, II and III) essential for its interaction with Nup120 and Nup85 that have been recently mapped in *Scer* using deletion analysis [43]. Domains I and II also overlap a 900 bp region that is the only essential fragment of the gene [44]. We examined Nup85 and showed that it strongly interacted with Nup145 and Nup120 in three species and their hybrids, suggesting that the inability of *Skud*-Nup145 to interact was unlikely affected by the DHFR fragment and that the effect was specific to its interaction with Nup120 (Figure 5*C*). We thus looked at whether amino acid differences in Nup145 between *Skud* and *Scer* could affect the three interaction domains. Because the yeast Nup145 protein is self-cleaved *in vivo* into two distinct but functional protein fragments, Nup145N and Nup145C [45], and because the DHFR fragments are fused to the C-terminus of proteins, we focused on the Nup145C fragment.

We found 81 amino acid (aa) changes unique to *Skud* Nup145C sequence (i.e. changes accumulated since the *Scer*-*Skud* divergence), with 27 of them located in three domains involved in Nup45C-Nup120 interaction [43], thus representing changes that could potentially affect the interaction (Figure S11 *A*). Among these changes, seven were concentrated in a very short region (13 aa length) of the main interaction domain (domain I) and resulted in three important aa polarity inversion (Figure S11 *B*). The fact that domain I is also located at the N-terminal end of Nup145C, that it concentrates several hypervariable regions, three phosphorylated sites [46], [47] and most of disordered regions of Nup145C [48], strongly suggests that this is the main interacting domain of the protein. Hence, several aa changes we observed in this domain between *Scer* and *Skud* are good candidates to explain the loss of interaction in *Skud*, since these changes could potentially enhanced a modification of the Nup145C N-terminal activity that subsequently caused this loss. We also found a 8 aa insertion at the C-terminal of the *Scer* Nup145, in the domain III (Figure S11), that may thus potentially affect the activity of this domain, while the absence of this insertion in *Skud* and *Suva* was not concordant with the absence of Nup145-Nup120 interaction in *Skud* only.

Interestingly, the interaction between Nup145C from *Suva* and Nup120 from *Skud* was also lost, as the interaction between Nup120 from *Suva* and Nup85 from *Skud* (Figure 5*C*–5*D*), suggesting that the architecture of this subcomplex, while conserved between *Scer* and *Suva*, may be modified in hybrids of *Suva* due to changes that occurred in this lineage. Many other variations in Nup145C sequence, like the 8 amino acids insertion in *Scer*, but also post-translational modifications and changes in regulation, may modulate these differences in PPIs we observed. Because Nup145 is an essential gene [37], we could expect that these interaction modifications would lead to incompatibilities in hybrids. However, we did not know whether the hybrid Nup145C-Nup120 interaction is essential for the cell, nor if incompatibilities between Nup145C and Nup120 actually affect the hybrid fitness, since parental proteins are all present in the diploid hybrid and thus parental interactions could occur and perform the function if hybrid interactions fail. In order to test if the loss of interactions could lead to an incompatibility between *Scer* and *Skud*, we constructed a haploid strain of *Scer* in which we replaced *Nup145* by its homologue from *Skud* (see Text S1). If the lack of interaction were causing an incompatibility, we would expect *Skud*-Nup145 to not be able to complement the loss of *Scer*-Nup145. Then we compared the relative growth of modified strains and wild types (WT) on enriched YPD medium, to directly test whether *SkudNup145* complemented *ScerNup145*. We observed a normal and similar growth for the WT and modified *Scer* strains, suggesting that, while essential, *Nup145* from *Scer*, can be complemented by *Nup145* from *Skud* (Figure 5*E*).

## Discussion

Investigating the role of protein-protein incompatibilities among closely related species is an underexplored but fundamental issue in evolutionary biology. Because interacting proteins are known to co-evolve within species [18]–[20], such incompatibilities are expected to accumulate as species diverge. Very few cases of such incompatibilities have been reported in the literature. One instance has recently been reported in the Proliferating Cell Nuclear Antigen (PCNA) protein complex between two groups of yeasts species [42]. However, the species investigated are distantly related (300 My), such that the question of whether protein incompatibilities can accumulate fast enough to result in inter-species incompatibilities during or just after a speciation event and thus cause inter-species incompatibilities is still an open one. Here, we adapted tools developed for the model species *Scer* to study PPIs *in vivo* in the closely related species *Skud*. We examined how two protein complexes with contrasting evolutionary properties could assemble in the hybrid between *Scer* and *Skud*. Given that the divergence time between *Skud* and *Scer* represents about 78% of the age of the *Saccharomyces* genus [23], with a molecular divergence range that is equivalent to molecular divergence between birds and mammals [22], *Saccharomyces* species cover the higher end of the spectrum of molecular divergence that can be observed among species that can be hybridized. As a result of this high divergence, F1 haploid recombinant hybrids of *Scer* and *Skud* are unviable [27], [28], while F1 diploid hybrids are viable [25], [26]. The inviability of F1 haploid hybrids makes the use of standard genetic mapping techniques to locate incompatible loci in the genome impossible. However, PCA allows us to examine potential incompatibilities resulting from PPIs perturbations directly in diploid F1 at the molecular and network levels.

Our results indicate that neither of our model protein complexes, the NPC nor the RNApII, is significantly perturbed in the hybrid between species. Our results are unintuitive since these two complexes have highly contrasting evolutionary histories in the two *Saccharomyces* species. *Scer*-*Skud* protein divergence within the RNApII never exceeds 3%, whereas nucleoporins diverge in the range of 7–41% (Figure 1), suggesting that unlike the RNApII where all proteins are essential for DNA transcription [35], the structure of the NPC is highly flexible [49]. The high conservation of PPIs is thus consistent with the evolutionary history of RNApII but not NPC. For instance, Nup82 is a key protein of the NPC that is involved in PPIs with almost all nucleoporins we tested. Despite the high protein divergence between *Scer* and *Skud* Nup82 (26%), we found that all these interactions were indistinguishable and highly correlated between species. This result likely illustrates that only few protein residues contribute to PPIs, as has been previously shown in many protein complexes of known structure [50]. In the NPC, very little detailed structural data is available, most of it from human [51], [52] and *Scer* [43], [53]. For instance, the Nup82-Nup116-Nup159 sub-complex structure was solved in *Scer* [53]. The Nup116-Nup82 interaction that we found to be the strongest in the NPC for *Scer* and *Skud* involves 23 Nup82 residues, among which 21 are conserved between these two species. This observation suggests that at least part of the robustness of protein complexes to inter-species hybridization results from the fact that few sites are involved and those are conserved, while the rest of the protein is free to evolve. The evolution of PPIs has recently been investigated for orthologous proteins in distant Eukaryotes [54]. While the high level of divergence between studied organisms restricted the number of investigable PPIs, these works provided strong evidences that PPIs evolve much slower than proteins, particularly for orthologous proteins which genes underwent no duplication event during their evolutionary history. Our works based on the *in vivo* detection of PPIs through a same method in two divergent species but with comparable level of organization, confirmed the high level of PPIs conservation at a lower scale of evolutionary time.

The few differences we observed for PPIs in hybrids were not significant in most cases (Figure 4), with the exception of one particular interaction occurring in *Scer* but not in *Skud* between nucleoporins Nup120 and Nup145C. By comparing PPIs signals in *Scer*, *Skud*, *Suva* and hybrids, we deduced that this interaction change was likely a lost in *Skud* rather than a gain in *Scer* (Figure 5*A*). Nup145C and Nup120 belong to a NPC heptameric sub-complex that has a partially known structure in *Scer* (Figure 5*B*). Fernandez-Martinez *et al.* showed that three Nup145C protein regions disrupted the Nup145C-Nup120 interaction when deleted [43]. Interestingly, one region that likely corresponds to the functional domain of Nup145C contains a high concentration of amino acids changes proper to *Skud*, potentially leading to substantial changes in the domain functionality (Figure S11), enforcing the hypothesis that these amino acid changes may had an impact on Nup145C interactions with other proteins. The complicated pattern of PPIs loss we observed in hybrids of *Suva* also suggests that the Nup145C-Nup120-Nup85 subcomplex accumulated several other differences in amino acids, in post-translational modifications or in protein regulation during *Suva* divergence with other *Saccharomyces* species (Figure 5*D*). Such changes, without affecting the functionality of the subcomplex in parental species, may increase the probability of protein incompatibilities to occur in hybrids. Our observations, based on evolutionary protein divergence, thus corroborate the work of Fernandez-Martinez *et al.* based on experimental protein domain mapping [43]. These results show that protein complexes such as the NPC diverge among closely related species. Because the tools we developed for the comparison of PINs among species are also suitable for high throughput screening of PPIs, they provide a powerful method to identify these changes at the whole interactome level.

The loss or gain of PPIs in hybrids as compared to parental species could potentially affect the functional organization of hybrid complexes, and thus lead to incompatibilities. However, PPI changes we found in the *Scer-Skud* hybrid were mostly associated with differences we already identified between parents, and were always congruent with the architecture of the complex. Because we demonstrated that the *Skud* gene coding for protein Nup145C, losing its interactions in hybrids, was interchangeable in function with its ortholog in *Scer* (Figure 5*E*), one can support that in this particular case, PPIs changes in hybrids had no effect on hybrid viability. It is interesting to note that the gene coding for the Nup145C is orthologous to Nup96 of *Drosophila simulans*, which was shown to be involved in male hybrid inviability by interacting with genes located on the X chromosome of *D. melanogaster* [55]. While the precise mechanism underlying this incompatibility is unknown, studies suggest that it likely occurs at the protein-protein interaction level, and that the strong coevolution acting on interacting nucleoporins drove the divergence between *D. melanogaster* and *D. simulans* Nup96 ortholog, the latter potentially becoming incapable of some vital interaction in the hybrid NPC [56].

Few genes were found to have a strong effect on yeast hybrid sterility [10] and because most of genetic incompatibilities are rather caused by interactions involving many genes [28], [57], [58], they potentially involve many PPI perturbations in hybrids. However, we found that two large protein complexes in yeasts *Skud* and *Scer* were highly robust to hybridization and thus unlikely to be involved in hybrid incompatibilities. Some works provided indirect evidence about the potential implication of PPIs loss in hybrids inviability due to variations found in natural populations of *S. cerevisiae* [59] and *S. paradoxus* [60], suggesting that even within-species protein variation is enough to generate PPIs perturbations in hybrids. *Skud* and *Scer* diverged 5–20 My ago and underwent contrasting evolutionary pressures. Further studies will be needed to examine other types of PPIs, such as smaller protein complexes and transient, signaling interactions. Incompatibilities may also accumulate in parts of PINs that underlie species-specific traits that would result from the contrasting ecological and metabolic characteristics of these two species: difference in growth temperature [61], in carbon source preference [62] and in alcohol metabolism [23]. The tools we developed here could first be used to identify these species-specific networks and then be harnessed to specifically address these questions.

## Methods

### Adaptation of the DHFR-PCA method to *S. paradoxus*, *S. kudriavzevii*, and *S. uvarum*

The DHFR-PCA method was developed for *Scer* strains BY4741 and BY4742 [13]. Haploid strains of other species used or constructed in this study are detailed in Table S3 and were constructed as described in Text S1, using oligonucleotides described in Table S4. In order to optimize PCA conditions for *Spar*, *Skud* and *Suva* and allow comparisons with *Scer*, we tested different growth conditions using control diploid strains able to produce a strong signal and with the same molecular interactions for the four species (Figure S1 *C*). In each species, we transformed the *MATa* and *MATα* strains with plasmids *p41-ZL-DHFR[1,2]* and *p41-ZL-DHFR[3]* respectively (construction detailed in Text S1). These plasmids express interacting leucine zipper moieties that strongly dimerize and thus lead to a strong signal in PCA. Negative controls consisted of plasmids expressing the linkers and DHFR fragments alone (*p41-Linker-DHFR[1,2]* and *p41-Linker-DHFR[3]*) [13] (Figure S1 *C*). We then tested different PCA conditions (temperature and methotrexate concentration) using spot dilution assays. Transformations, crosses, diploid selection and PCA were performed as described in Text S1. Plates were manually analyzed with Adobe Photoshop from digital images and cell growth was estimated from pixel intensities of spot dilutions after six days of incubation (Figure S12).

### Construction of strains for the DHFR-PCA in the NPC and RNApII complexes

All *Skud MATa* (FM1109) and *Skud MATα* (FM1110) strains for PPI screening (RNApII, 9 proteins; NPC, 15 proteins; see Figure S1A and Text S1 for details of constructions) were constructed by homologous recombination, by using primers described in Table S5. We completed the DHFR collection for *Scer* [13] since some strains were missing for these complexes and/or incorrectly tagged (Table S1). We confirmed all strains by PCR-sequencing across the 3′ end of the coding region of the genes and the fragment of the DHFR-PCA cassette encompassing the entire linker and the 5′ region of the DHFR fragments. All strains were reconfirmed by PCR a second time before screening and those that did not show PCR amplification were discarded.

### Screening of PPIs in the NPC and RNApII complexes

We performed all possible crosses among the 24 *MATa*-DHFR F[1,2] and 24 *MATα*-DHFR F[3] strains constructed and confirmed for the NPC and the RNApII for a maximum of 576 pairwise combinations per species. We additionally made all possible hybrid crosses between *Scer* and *Skud*, given two types of hybrid diploids per combination: hybrid 1 (*MATa Scer* crossed with *MATα Skud*) and hybrid 2 (*MATa Skud* crossed with *MATα Scer*). Each possible combination was independently repeated three times (triplicates) on the same plate at random positions (Figure S13). Haploid strains were crossed on solid YPD medium and resulting diploid strains were selected two successive times on YPD with antibiotics using a robot-handled pin tool (see Text S1 and Figure S13). Diploid cells were transferred onto 1536-array plates of solid synthetic medium with methotrexate 200 µg/mL and incubated for five days at 30°C. Digital image analyses were performed using a custom script implemented in the software ImageJ 1.45 m (http://rsbweb.nih.gov/ij/).

### Correction of SI values and regression analysis

Uncorrected SI values measured in species were highly correlated to those measured in hybrids (r = 0.87 to 0.91, *p*<0.001; Figures S4, S5, S6, S7). However, SI values associated to a particular protein in a species were sometimes biased between *MATa* and *MATα* strains. For instance, SI values associated to proteins Rpb8 and Nsp1 of *Scer* tagged in *MATα* were always lower than SI measured in *MATa* (Figures S4 and S6), whereas we observed the reverse for protein Rpb10 (Figures S5 and S7). In most of regressions tested, SI values associated to a protein tagged in a mating-type remained significantly correlated between species and hybrid, while differing from SI values associated to the opposed mating-type. We thus performed a correction of SI values by considering independently each protein tagged in a particular strain (Figure S2 *B*–S2*F*). For each set of SI values associated to a haploid strain, we performed a correction only if the correlation species/hybrid was significant (Figure S2 *C*), which was not the case for only two comparisons (Figure S7 *A*). Then, we fitted the regression so that mean SI was equal in species and hybrid (Figure S2 *D*). Corrections could be realized in two different ways: by increasing SI for biased strains, with the risk to increase the signal background, or by decreasing SI for unbiased strains, with the risk to lose PPI signals in the background. We choose the second way, which was the most conservative one. Then we grouped all corrected SI values together (Figure S2 *E*) and obtained a significant increase of correlations quality for comparisons between species and hybrids (r = 0.94 to 0.97, *p*<0.001; Figures S4, S5, S6, S7). For downstream analysis, we considered that a SI value was different between species and hybrid or among species when the associated residual value significantly deviate from the distribution of all residual values grouped together (Figure S2 *F*).

## Supporting Information

Dataset S1 Contains raw SI values for *Scer*, *Skud*, hybrids 1 (*Scer MATa*×*Skud MATα*) and hybrid 2 (*Skud MATa*×*Scer MATα*).
(XLSX)

Dataset S2 Contains SI values corrected for comparisons A) *Scer vs.* Hybrid 2 (protein of interest tagged in *MATa*); B) *Scer vs.* Hybrid 1 (protein of interest tagged in *MATα*); C) *Skud vs.* Hybrid 1 (protein of interest tagged in *MATa*); D) *Skud vs.* Hybrid 2 (protein of interest tagged in *MATα*); E) *Scer vs. Skud*.
(XLSX)

Figure S1 Principle of the DHFR-PCA in yeasts. The DHFR-PCA screen is based on the resistance of strains to methotrexate (MTX) provided by an engineered mouse dihydrofolate reductase enzyme (DHFR). The DHFR consists of two complementary protein fragments DHFR[1,2] and DHFR[3], reconstituting the DHFR enzyme that is insensitive to MTX. (*A*) Construction of haploid *MATa* and *MATα* strains to fuse two genes *Gene1* and *Gene2* with, respectively, cassettes *DHFR[1,2]-NatMX4* and *DHFR[3]* *-HPH*. Cassettes were amplified by PCR from plasmids *pAG25-DHFR[1,2]* and *pAG32-DHFR[3]* with forward primers G1-5′ and G2-5′ and reverse primers G1-3′ and G2-3′, and were incorporated at the 3′ end of the targeted gene by homologous recombination. The resulting fusion proteins, P1 and P2, were respectively fused to the DHFR[1,2] (*MATa*) or the DHFR[3] (*MATα*) protein fragment via a flexible linker. (*B*) In diploid cells, the DHFR activity is recovered if P1 and P2 interact, so that the interaction could be detected according to strain growth on medium with MTX. (*C*) Construction of control diploid strains for DHFR-PCA optimization in different *Saccharomyces* species. Haploid *MATa* and *MATα* strains were transformed with, respectively, plasmid *p41-linker-DHFR[1,2]* and *p41-linker-DHFR[3]* and crossed to produce a negative control diploid strain in which DHFR fragments were unable to complement (top); or with, respectively, plasmids *p41-zipper-linker-DHFR[1,2]* (*p41-ZL-DHFR[1,2]*) and *p41-zipper-linker-DHFR[3]* (*p41-ZL-DHFR[3]*) allowing the complementation of DHFR fragments via the strong interaction between two GCN4 parallel coiled-coil leucine zipper fragments, restituting the cell resistance to MTX (bottom).
(PDF)

Figure S2 Regression analysis of SI signals measured in *Scer*, *Skud*, hybrid 1 (*Scer MATa* crossed with *Skud MATα*) and hybrid 2 (*Skud MATa* crossed with *Scer MATα*): example of PPIs screening between four hypothetical proteins. (*A*) Comparisons of SI values between species and two hybrids measured for four hypothetical proteins (P1 to P4). Circles and squares represent respectively the protein tagged in *MATa* (with DHFR F[1,2] fragment) or in *MATα* (with DHFR F[3] fragment). In order to evaluate the conservation of a PPI between *Scer* and hybrids, the SI value measured in *Scer* was compared with that measured (1) in hybrid 2 (protein of interest tagged in *MATa*) or (2) in hybrid 1 (protein of interest tagged in *MATα*). In order to evaluate the conservation of a PPI between *Skud* and hybrids, the SI value measured in *Skud* was compared with that measured (3) in hybrid 1 (protein of interest tagged in *MATa*) or (4) in hybrid 2 (protein of interest tagged in *MATα*). (*B*) Raw SI data in four types of comparisons are poorly correlated mostly because of (*C*) variation in SI intensities when the protein of interest is tagged in the *MATa* (1–3) or in the *MATα* strain (2–4), while SI values for *MATa* and *MATα* taken alone are highly correlated between species and hybrids. (*D*) Correction of SI values according to the regression between species and hybrids if the correlation is significant for the protein of interest. (*E*) Corrected SI values are pooled together. Divergent interactions appear as outliers (orange arrows) and (*F*) could be tested according to the distribution of all residual SI values pooled together.
(PDF)

Figure S3 Comparisons of normalized colony size (log_10_) measured from 1536-arrays after 5 days of growth on methotrexate medium. For each array (*Scer*: red; *Skud*: blue; hybrids: green), replicates 1 and 2 (left), 1 and 3 (center) and 2 and 3 (right) were compared. Correlations were tested using a Pearson's correlation test (*p*<0.001).
(PDF)

Figure S4 Regression analysis of SI values between *Scer* and hybrid 2 (protein of interest tagged in *MATa*). Black circles indicate raw SI values. Red points indicate corrected SI values. (*A*) Comparison of SI values in each protein of NPC taken independently. (*B*) Comparison of SI values in each protein of RNApII taken independently. (C) Comparison of all SI values pooled together. Correlations were tested before (black) and after correction (red) using a Pearson's correlation test (*p*<0.001).
(PDF)

Figure S5 Regression analysis of SI values between *Scer* and hybrid 1 (protein of interest tagged in *MATα*). Black circles indicate raw SI values. Red points indicate corrected SI values. (*A*) Comparison of SI values in each protein of NPC taken independently. (*B*) Comparison of SI values in each protein of RNApII taken independently. (C) Comparison of all SI values pooled together. Correlations were tested before (black) and after correction (red) using a Pearson's correlation test (*p*<0.001). Unavailable strains are indicated in grey.
(PDF)

Figure S6 Regression analysis of SI values between *Skud* and hybrid 1 (protein of interest tagged in *MATa*). Black circles indicate raw SI values. Blue points indicate corrected SI values. (*A*) Comparison of SI values in each protein of NPC taken independently. (*B*) Comparison of SI values in each protein of RNApII taken independently. (C) Comparison of all SI values pooled together. Correlations were tested before (black) and after correction (blue) using a Pearson's correlation test (*p*<0.001).
(PDF)

Figure S7 Regression analysis of SI values between *Skud* and hybrid 2 (protein of interest tagged in *MATα*). Black circles indicate raw SI values. Blue points indicate corrected SI values. (*A*) Comparison of SI values in each protein of NPC taken independently. (*B*) Comparison of SI values in each protein of RNApII taken independently. (C) Comparison of all SI values pooled together. Correlations were tested before (black) and after correction (blue) using a Pearson's correlation test (*p*<0.001). Unavailable strains are indicated in grey.
(PDF)

Figure S8 Comparison of SI values after correction by regression analysis between *Scer* and hybrid 2 (left) or hybrid 1 (right). (*A*) Estimation of the SI threshold value (*t*) for the detection of PPIs in *Scer*. (*B*) Estimation of the SI threshold value (*t*) for the detection of PPIs in hybrids. In each figure, distribution of SI values (log_10_) is showed with solid lines representing SI values measured within complexes (SW values) and dotted lines representing SI values measured among complexes (SA values). The *t* value corresponds to the maximal SA value measured in the diploid. Grey frames indicate background growth (SI<*t*). (*C*) Overlapped networks of *Scer* and hybrids Only SI above *t* and comparable interactions are represented. Line width is proportional to SI values measured between proteins in *Scer* (red) and hybrids (green) in the NPC (left) and the RNApII (right). Interactions appear in brown when *Scer* and hybrid SI values overlap. Different degrees of brown depend on whether the interaction could be tested in reciprocal ways or not.
(PDF)

Figure S9 Comparison of SI values after correction by regression analysis between *Skud* and hybrid 1 (left) or hybrid 2 (right). (*A*) Estimation of the SI threshold value (*t*) for the detection of PPIs in *Skud*. (*B*) Estimation of the SI threshold value (*t*) for the detection of PPIs in hybrids. In each figure, distribution of SI values (log_10_) is shown with solid lines representing SI measured within complexes (SW values) and dotted lines representing SI measured among complexes (SA values). The *t* value corresponds to the maximal SA value measured in the diploid. Grey frames indicate background growth (SI<*t*). (*C*) Overlapped networks of *Skud* and hybrids Only SI above *t* and comparable interactions are represented. Line width is proportional to SI values measured between proteins in *Skud* (blue) and hybrids (green) in the NPC (left) and the RNApII (right). Interactions appear in turquoise when *Skud* and hybrid SI values overlap. Different degrees of turquoise depend in whether the interaction could be tested in reciprocal ways or not.
(PDF)

Figure S10 (*A*) Venn diagram showing the overlap of PPIs in *Scer* for NPC and RNApII identified by other physical methods than PCA (BioGRID). (*B*) Plot of 82 SW values corresponding to 54 PPIs we identified by PCA in *Scer*, against the number of occurrences of the PPI in BioGRID (Pearson's correlation; *p*<0.001). (*C*) Schematic representation of the RNApII complex based on crystal structure [35]. Protein names are located at the approximate position of the C-terminal of each protein. Lines indicate interactions detected only in *Scer* (red), only in *Skud* (blue) or in both species (purple). Dotted lines indicate interactions only detected in *Scer* because the information was not available in *Skud*. Bold lines indicate PPIs that were identified in reciprocal combinations. Only PPIs with SW>*t* are showed. The Rpc10 C-terminal is hidden by other proteins and Rpb9 is located at the opposite side of the complex, which was in agreement of the absence of PPIs for these proteins. (*D*) Schematic representation or the NPC organization based on structural data [34].
(PDF)

Figure S11 Amino acid (aa) divergence between *Scer*, *Skud* and *Suva* for the Nup145C protein. (*A*) Distribution of aa changes along the Nup145C protein (proportion of aa changes in a window of 9 aa). Changes filled in blue are unique to *Skud*. Domains I, II and III involved in the Nup145C-Nup120 interaction are indicated by brackets. Red, blue and yellow bars indicated disordered regions of the protein in respectively *Scer*, *Skud* and *Suva*, predicted by DISOPRED2 [63]. Grey and purple arrows indicate locations of respectively a high concentration of aa changes proper to *Skud* in domain I and a 8 aa insertion proper to *Scer* in domain III. (*B*) Detail of the aa divergence between three species in domains I, II and III with *Scer* as reference sequence. Dashes indicate no aa change; asterisks indicate deletions. Changes proper to *Skud* are indicated in blue. Green and orange positions indicate respectively positive and negative aa polarity changes in *Skud*. Phosphorylation sites are indicated by circled P. Grey and purple frames indicate locations of respectively the high concentration of aa changes specific to *Skud* in domain I and the 8 aa insertion proper to *Scer* in domain III. Predicted disordered regions are also indicated.
(PDF)

Figure S12 Optimization of the DHFR-PCA in four *Saccharomyces* species. Difference in growth among positive (zipper-linker) and negative (linker) controls on methotrexate (MTX) tested in each species and each condition: MTX concentration (columns), incubation temperature and culture OD_600_ (rows). Each box represents a combination of conditions within a species (three replicates for each combination). The grey scale is proportional to the difference in relative spot growth (averaged among three replicates) between positive and negative controls (scale on bottom). In each condition, differences were tested using a t-test (***: *p*<0.001; **: *p*<0.01; *: *p*<0.05; n.s.: *p*>0.05).
(PDF)

Figure S13 Design of the DFHR-PCA screen for the NPC and RNApII complexes. For each species and hybrids, *MATa*-DHFR[1,2] strains (top left) were incubated to saturation in 96-position pre-culture plates and divided in three parts (dotted lines), each containing one replicate of one of 24 *MATa*-DHFR[1,2] strains positioned at random. Cultures were printed four times with a 96-pin tool on a 86×128 mm plate (omnitray) filled with 35 ml of solid YPD medium with nourseothricin (100 mg/L), to obtain a 384-positions array. For each *MATα*-DHFR[3] strain (top right), an empty omnitray was filled with 20 ml of a fresh saturated liquid YPD culture and cells were transferred with a 96-position pin-tool on a plate with 35 ml of solid YPD and hygromycin B (250 mg/L). Four *MATα* strains were positioned per omnitray in order to obtain an interlaced array of 384 positions. 384-plates of *MATa* and *MATα* strains were crossed using a 384-pin tool on an omnitray with 35 ml of solid YPD. After incubation, colonies were transferred onto a 1536-array omnitray with 35 ml on solid YPD with nourseothricin and hygromycin B to allow diploid selection. Then, cells were transferred onto an omnitray filled with 35 ml of solid synthetic medium without adenine and with 2% methotrexate.
(PDF)

Table S1 List of DHFR-PCA strains used in this study.
(DOCX)

Table S2 Summary of testable PPIs in *Scer*, *Skud* and their hybrids. Details of calculation are given under the table. Codes for different testable combinations of PPIs: S: homomeric interactions (P1-P1); NR: non-reciprocal interactions (only P1-P2 or P2-P1 is testable); R: reciprocal interactions (both P1-P2 and P2-P1 are testable).
(DOCX)

Table S3 Original strains used in this study.
(DOCX)

Table S4 List of oligonucleotides used in this study.
(DOCX)

Table S5 List of oligonucleotides used for the DHFR-PCA strain construction.
(DOCX)

Text S1 Supplementary methods.
(DOCX)
