# Supplementary figures and images for: Evidence for the Robustness of Protein Complexes to Inter-Species Hybridization
Source: PLoS Genet. 2012 Dec 27;8(12):e1003161. doi: 10.1371/journal.pgen.1003161 (PMC3531474; doi:10.1371/journal.pgen.1003161)

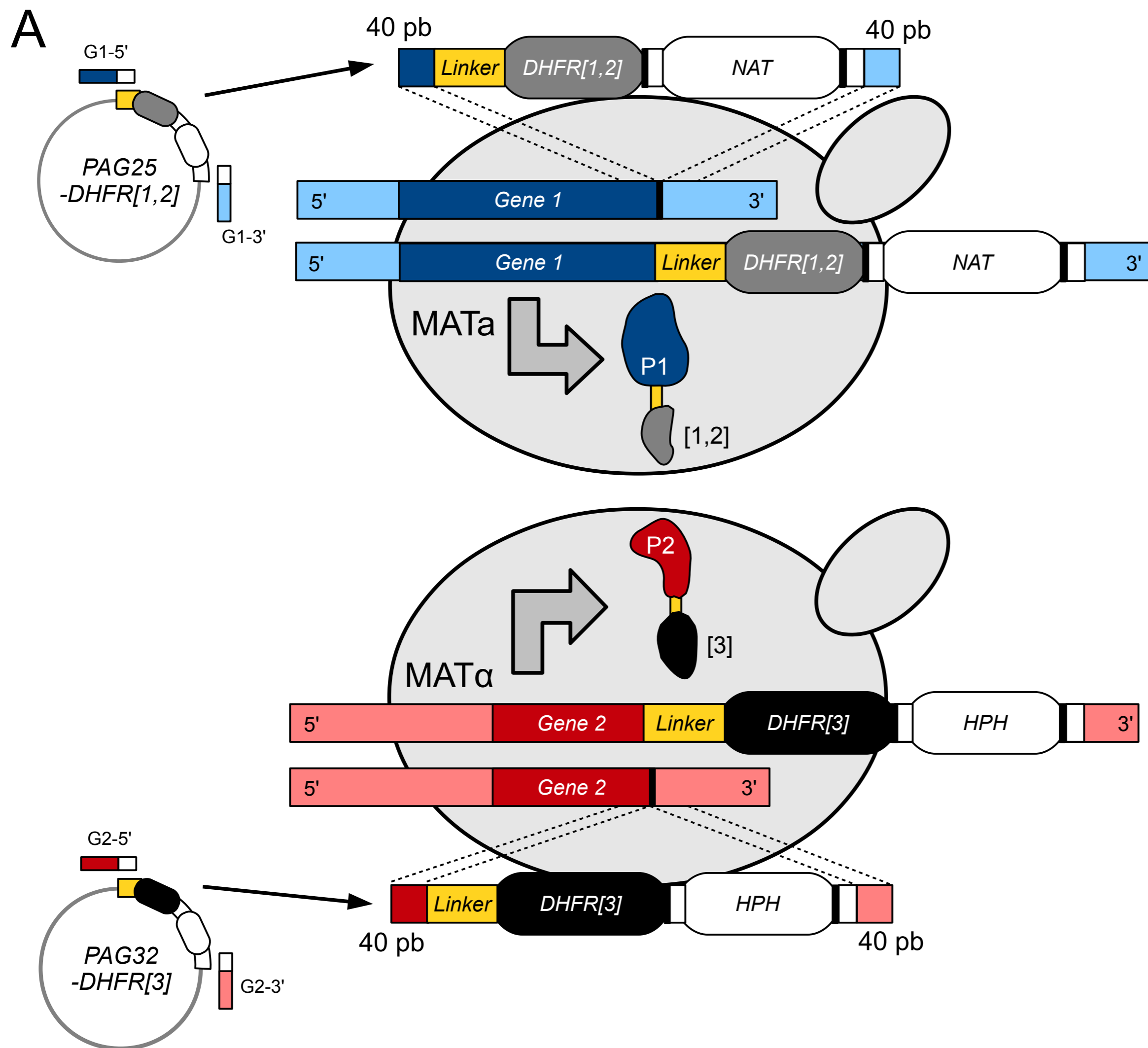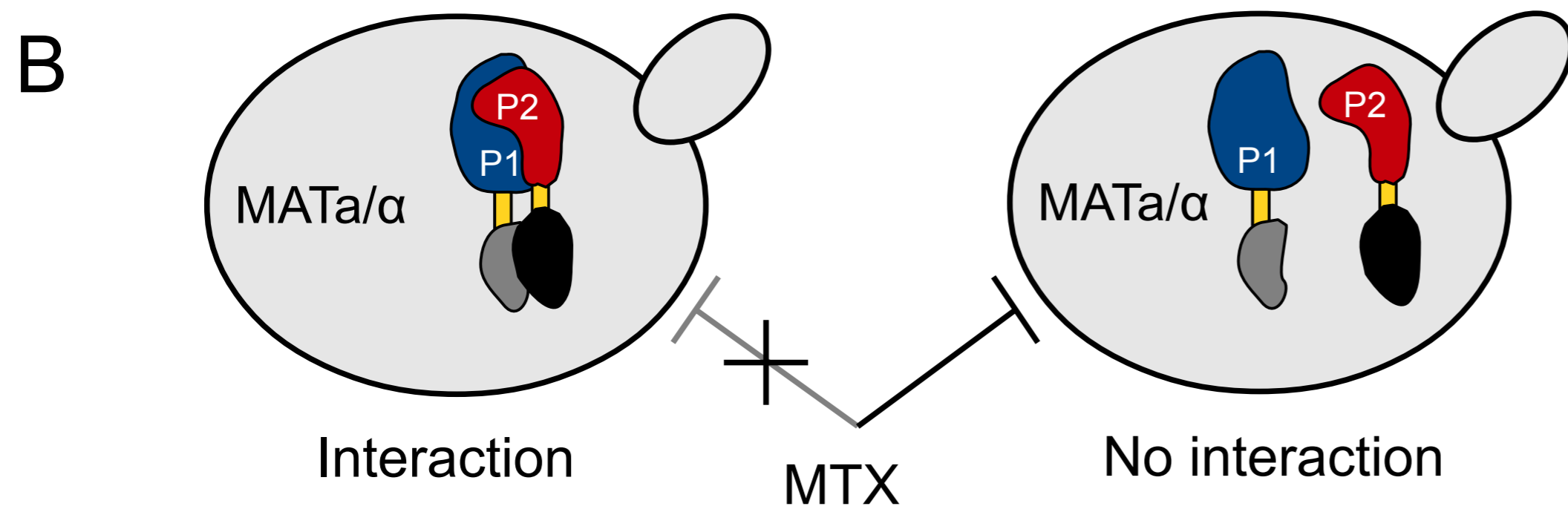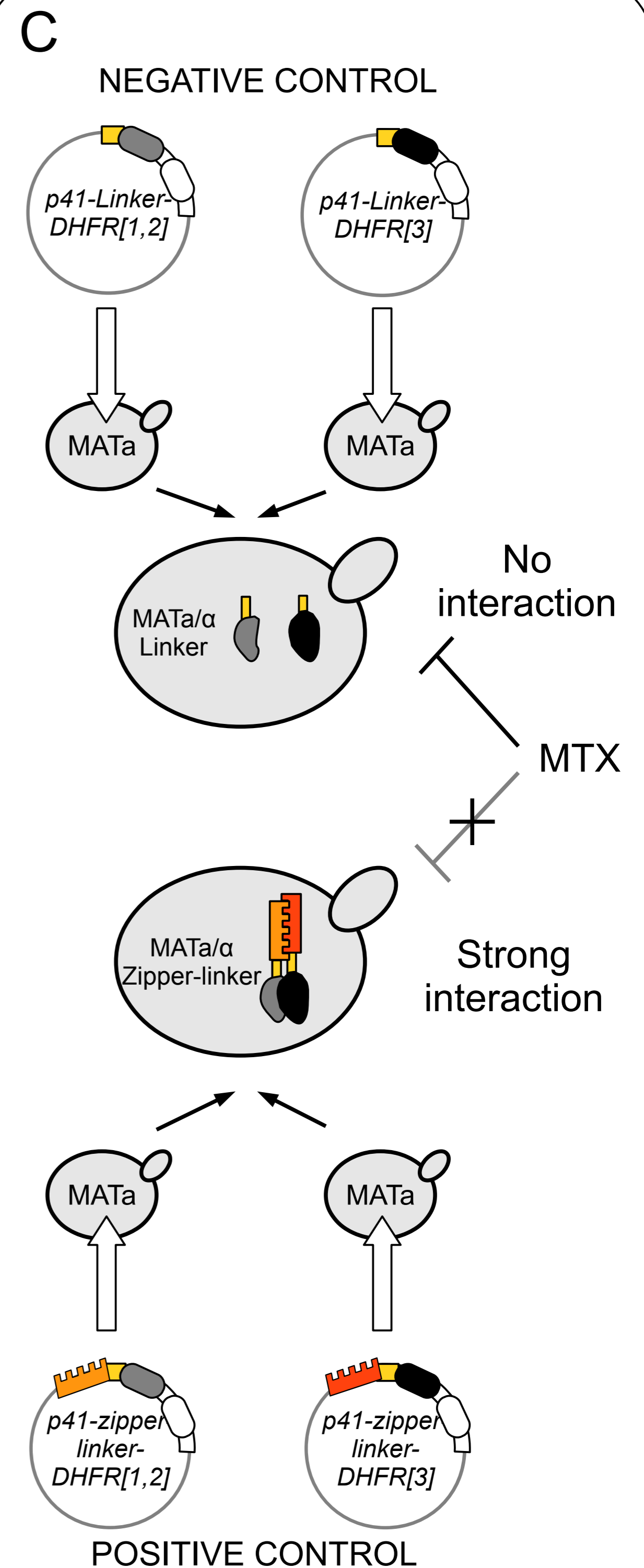

Supplement: Figure S1 — Principle of the DHFR-PCA in yeasts. The DHFR-PCA screen is based on the resistance of strains to methotrexate (MTX) provided by an engineered mouse dihydrofolate reductase enzyme (DHFR). The DHFR consists of two complementary protein fragments DHFR[1,2] and DHFR[3], reconstituting the DHFR enzyme that is insensitive to MTX. (A) Construction of haploid MATa and MATα strains to fuse two genes Gene1 and Gene2 with, respectively, cassettes DHFR[1,2]-NatMX4 and DHFR[3] -HPH. Cassettes were amplified by PCR from plasmids pAG25-DHFR[1,2] and pAG32-DHFR[3] with forward primers G1-5′ and G2-5′ and reverse primers G1-3′ and G2-3′, and were incorporated at the 3′ end of the targeted gene by homologous recombination. The resulting fusion proteins, P1 and P2, were respectively fused to the DHFR[1,2] (MATa) or the DHFR[3] (MATα) protein fragment via a flexible linker. (B) In diploid cells, the DHFR activity is recovered if P1 and P2 interact, so that the interaction could be detected according to strain growth on medium with MTX. (C) Construction of control diploid strains for DHFR-PCA optimization in different Saccharomyces species. Haploid MATa and MATα strains were transformed with, respectively, plasmid p41-linker-DHFR[1,2] and p41-linker-DHFR[3] and crossed to produce a negative control diploid strain in which DHFR fragments were unable to complement (top); or with, respectively, plasmids p41-zipper-linker-DHFR[1,2] (p41-ZL-DHFR[1,2]) and p41-zipper-linker-DHFR[3] (p41-ZL-DHFR[3]) allowing the complementation of DHFR fragments via the strong interaction between two GCN4 parallel coiled-coil leucine zipper fragments, restituting the cell resistance to MTX (bottom). (PDF) [file pgen.1003161.s003.pdf]

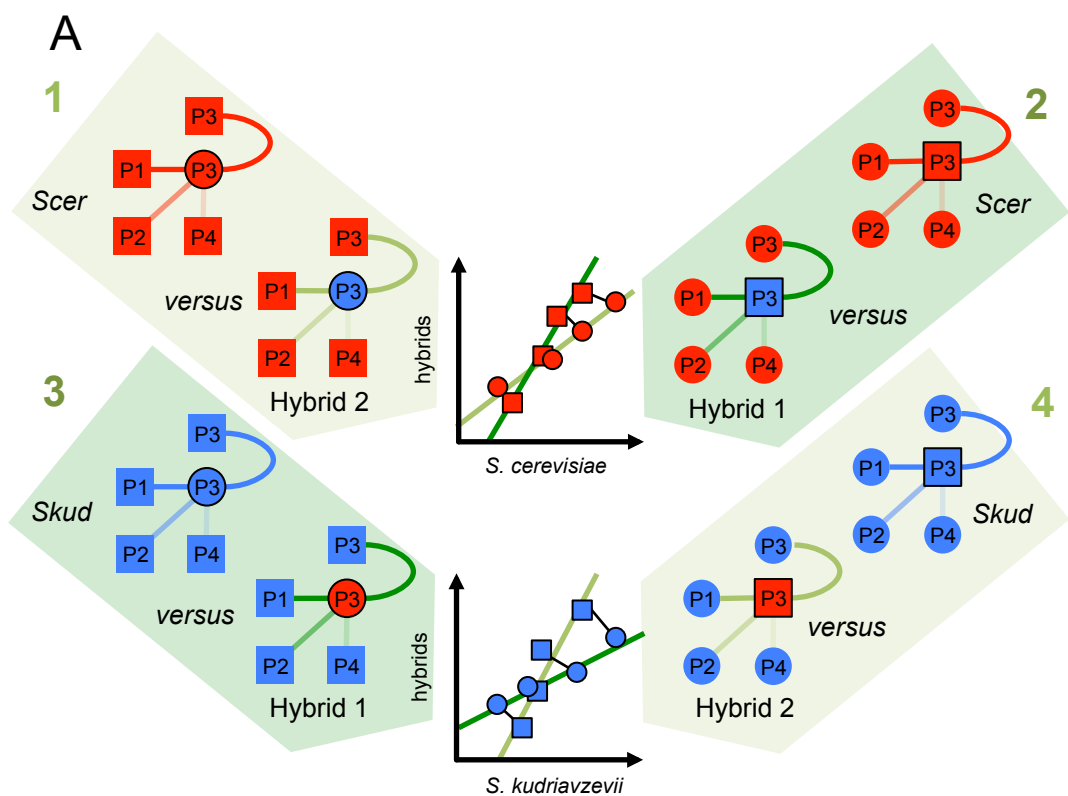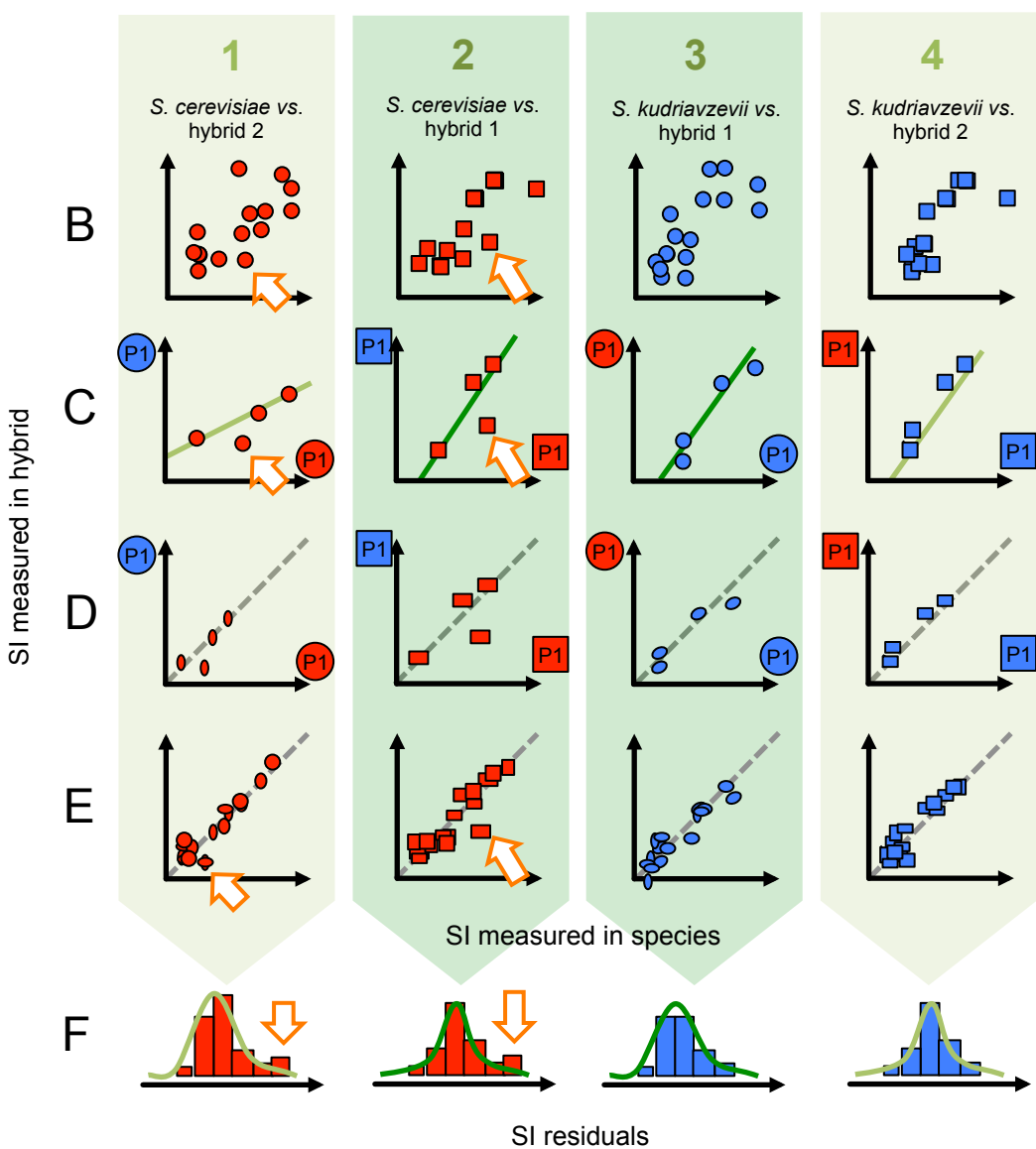

Supplement: Figure S2 — Regression analysis of SI signals measured in Scer, Skud, hybrid 1 (Scer MATa crossed with Skud MATα) and hybrid 2 (Skud MATa crossed with Scer MATα): example of PPIs screening between four hypothetical proteins. (A) Comparisons of SI values between species and two hybrids measured for four hypothetical proteins (P1 to P4). Circles and squares represent respectively the protein tagged in MATa (with DHFR F[1,2] fragment) or in MATα (with DHFR F[3] fragment). In order to evaluate the conservation of a PPI between Scer and hybrids, the SI value measured in Scer was compared with that measured (1) in hybrid 2 (protein of interest tagged in MATa) or (2) in hybrid 1 (protein of interest tagged in MATα). In order to evaluate the conservation of a PPI between Skud and hybrids, the SI value measured in Skud was compared with that measured (3) in hybrid 1 (protein of interest tagged in MATa) or (4) in hybrid 2 (protein of interest tagged in MATα). (B) Raw SI data in four types of comparisons are poorly correlated mostly because of (C) variation in SI intensities when the protein of interest is tagged in the MATa (1–3) or in the MATα strain (2–4), while SI values for MATa and MATα taken alone are highly correlated between species and hybrids. (D) Correction of SI values according to the regression between species and hybrids if the correlation is significant for the protein of interest. (E) Corrected SI values are pooled together. Divergent interactions appear as outliers (orange arrows) and (F) could be tested according to the distribution of all residual SI values pooled together. (PDF) [file pgen.1003161.s004.pdf]

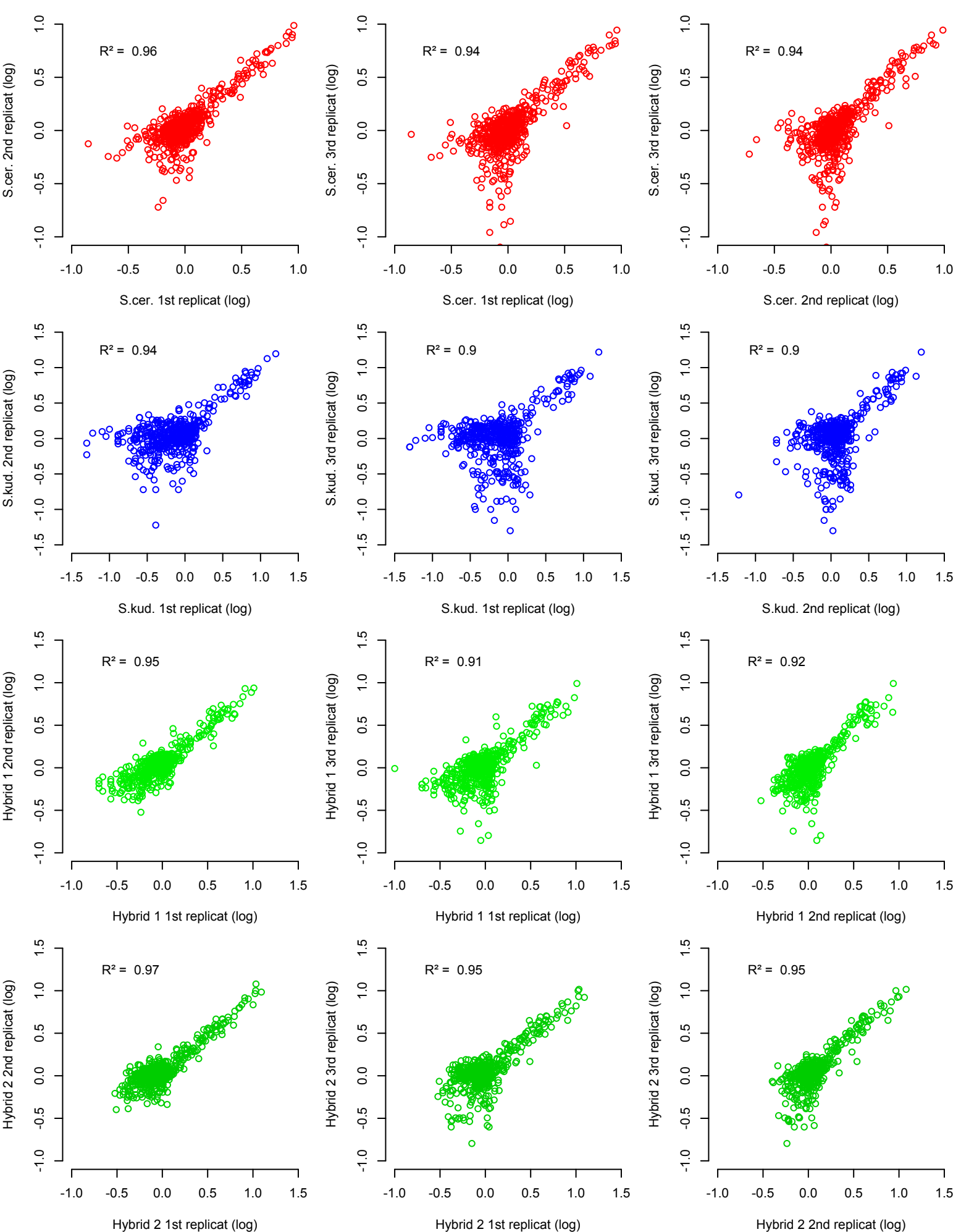

Supplement: Figure S3 — Comparisons of normalized colony size (log10) measured from 1536-arrays after 5 days of growth on methotrexate medium. For each array (Scer: red; Skud: blue; hybrids: green), replicates 1 and 2 (left), 1 and 3 (center) and 2 and 3 (right) were compared. Correlations were tested using a Pearson's correlation test (p<0.001). (PDF) [file pgen.1003161.s005.pdf]

A

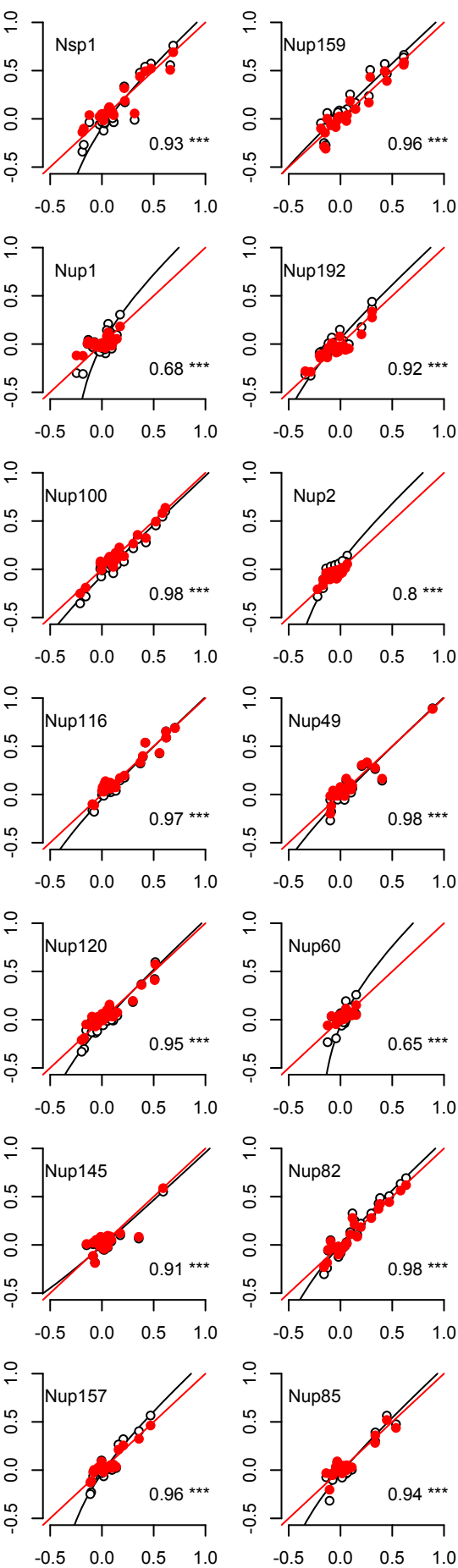

B

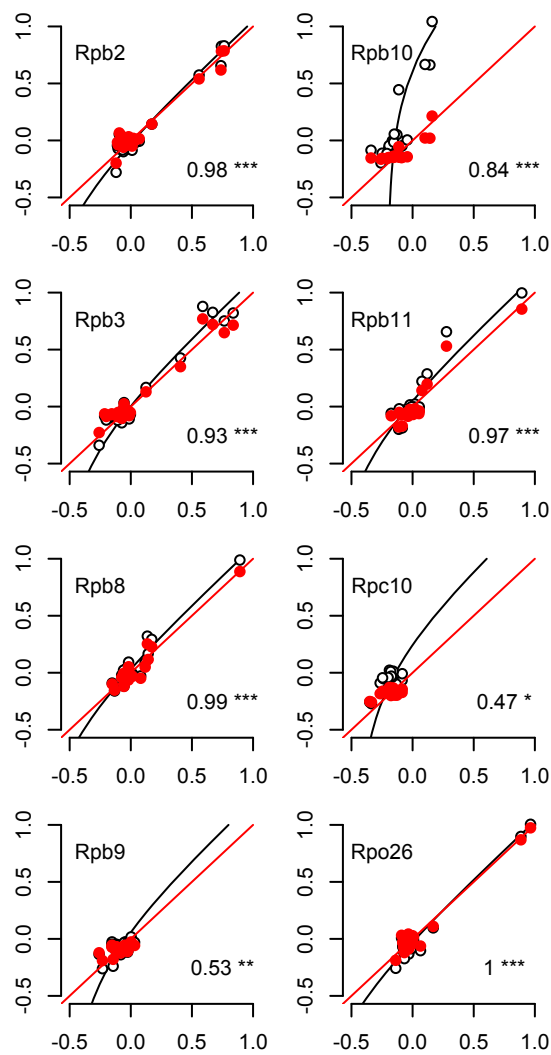

C

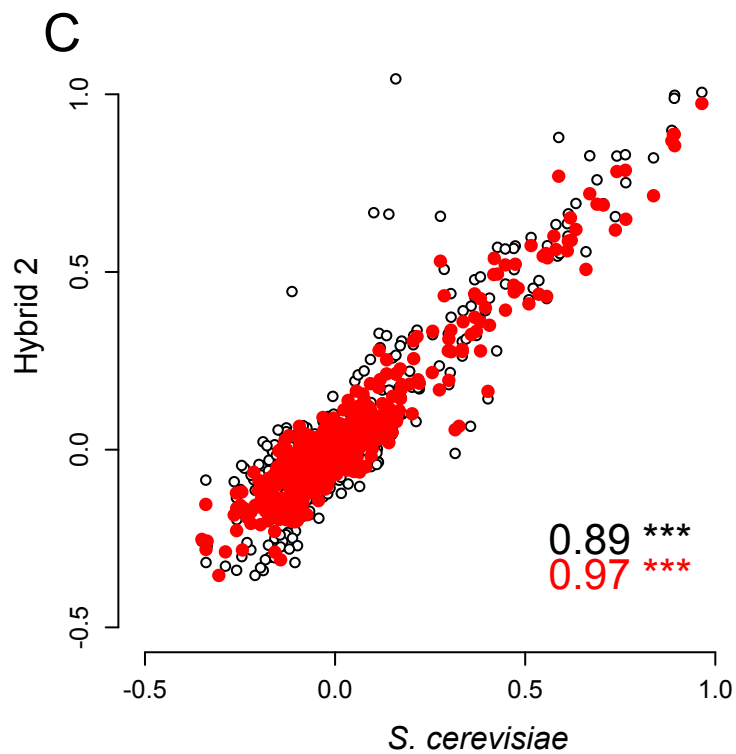

Supplement: Figure S4 — Regression analysis of SI values between Scer and hybrid 2 (protein of interest tagged in MATa). Black circles indicate raw SI values. Red points indicate corrected SI values. (A) Comparison of SI values in each protein of NPC taken independently. (B) Comparison of SI values in each protein of RNApII taken independently. (C) Comparison of all SI values pooled together. Correlations were tested before (black) and after correction (red) using a Pearson's correlation test (p<0.001). (PDF) [file pgen.1003161.s006.pdf]

A

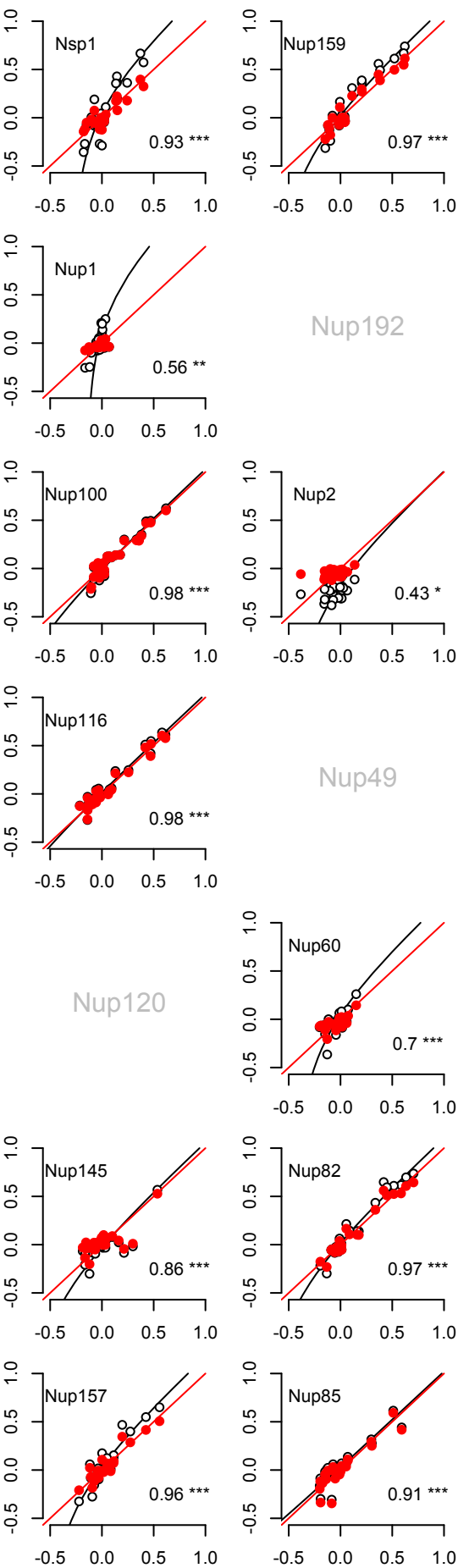

B

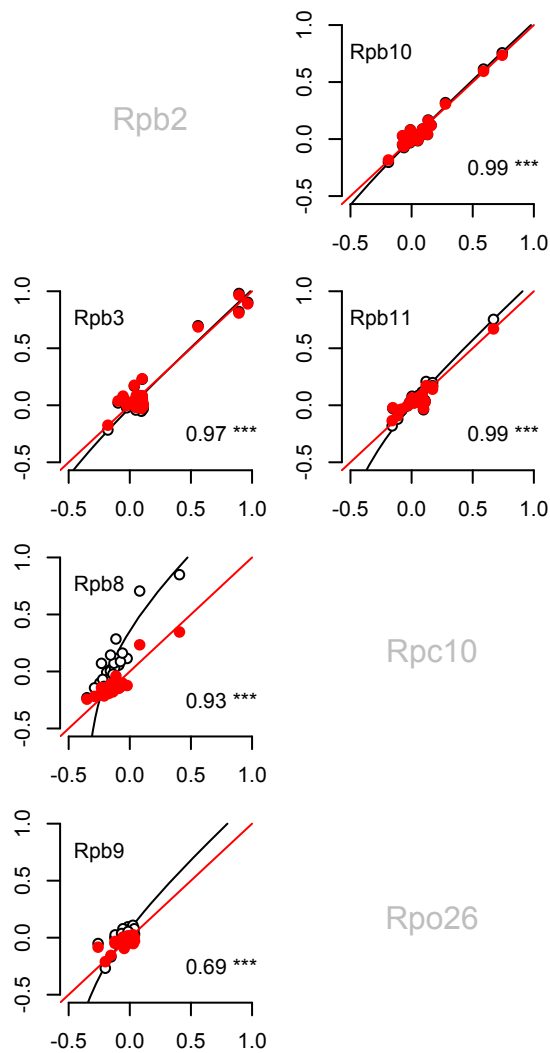

C

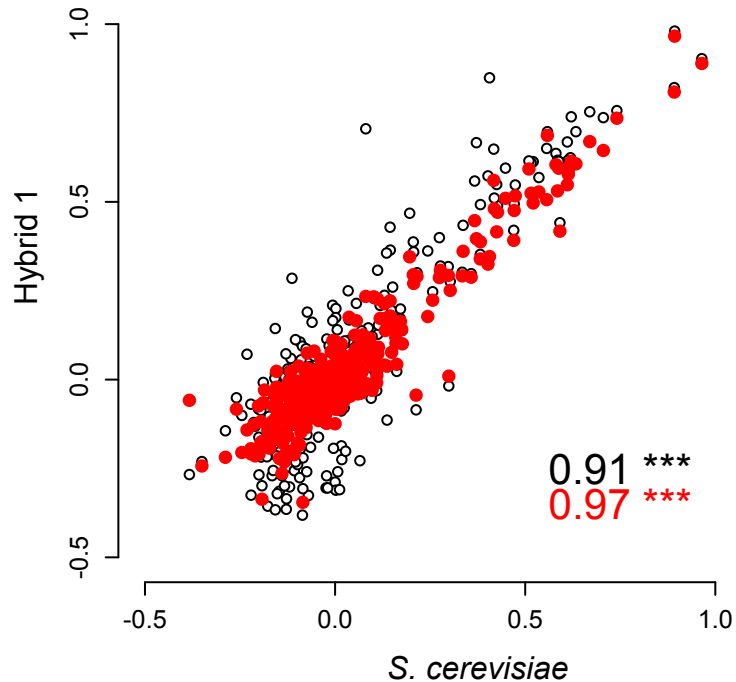

Supplement: Figure S5 — Regression analysis of SI values between Scer and hybrid 1 (protein of interest tagged in MATα). Black circles indicate raw SI values. Red points indicate corrected SI values. (A) Comparison of SI values in each protein of NPC taken independently. (B) Comparison of SI values in each protein of RNApII taken independently. (C) Comparison of all SI values pooled together. Correlations were tested before (black) and after correction (red) using a Pearson's correlation test (p<0.001). Unavailable strains are indicated in grey. (PDF) [file pgen.1003161.s007.pdf]

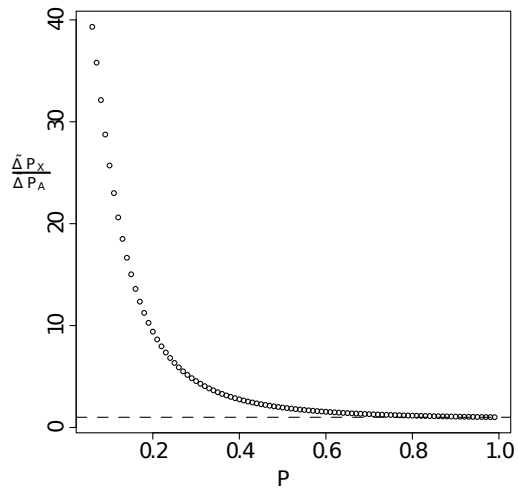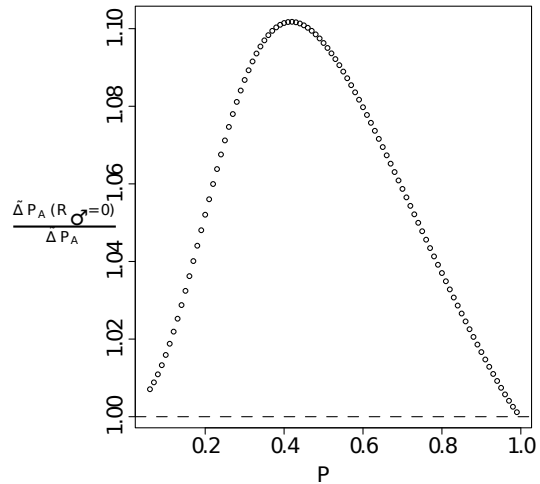

Supplement: Figure S6 — Regression analysis of SI values between Skud and hybrid 1 (protein of interest tagged in MATa). Black circles indicate raw SI values. Blue points indicate corrected SI values. (A) Comparison of SI values in each protein of NPC taken independently. (B) Comparison of SI values in each protein of RNApII taken independently. (C) Comparison of all SI values pooled together. Correlations were tested before (black) and after correction (blue) using a Pearson's correlation test (p<0.001). (PDF) [file pgen.1003161.s008.pdf]

A

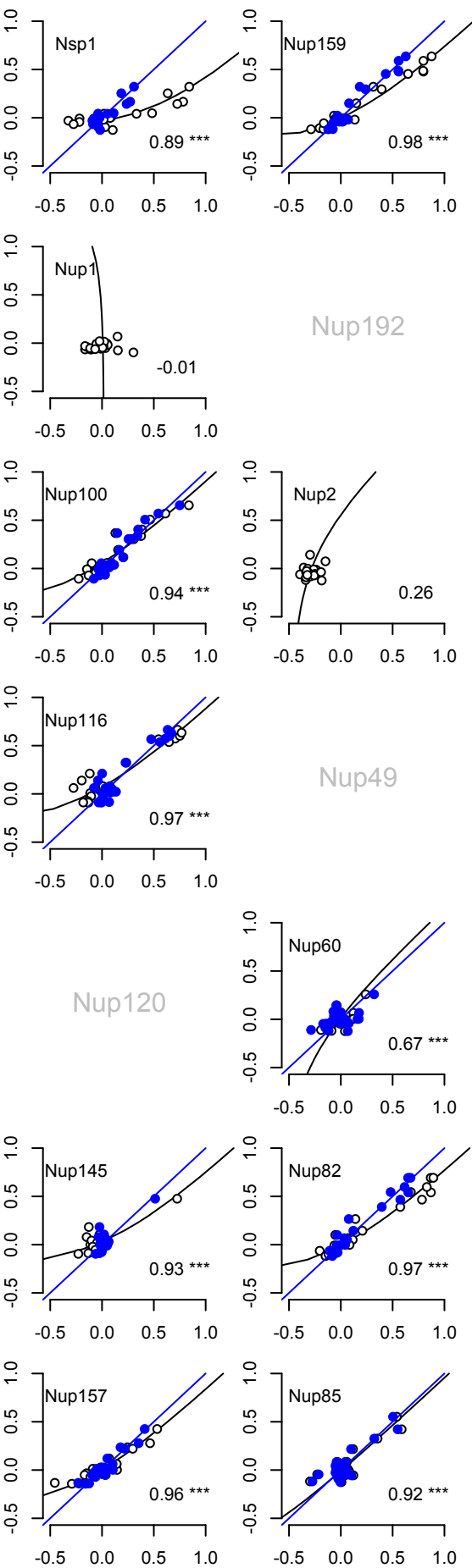

B

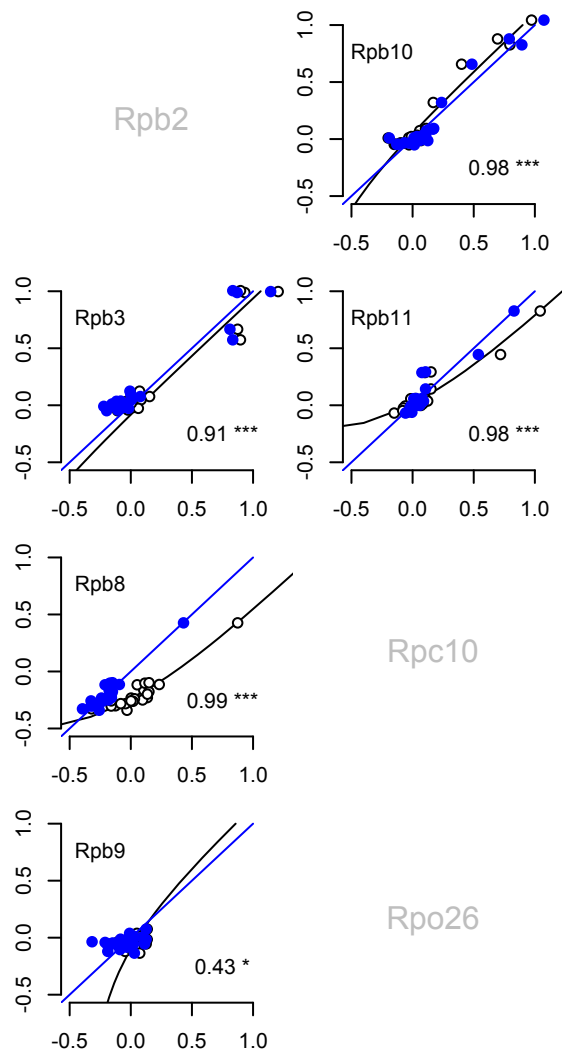

C

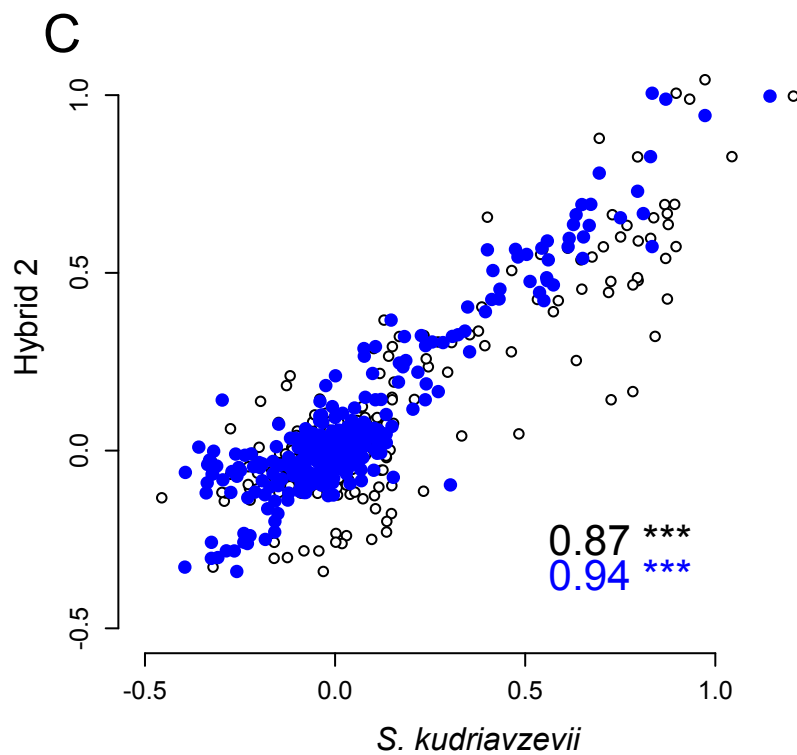

Supplement: Figure S7 — Regression analysis of SI values between Skud and hybrid 2 (protein of interest tagged in MATα). Black circles indicate raw SI values. Blue points indicate corrected SI values. (A) Comparison of SI values in each protein of NPC taken independently. (B) Comparison of SI values in each protein of RNApII taken independently. (C) Comparison of all SI values pooled together. Correlations were tested before (black) and after correction (blue) using a Pearson's correlation test (p<0.001). Unavailable strains are indicated in grey. (PDF) [file pgen.1003161.s009.pdf]

**A**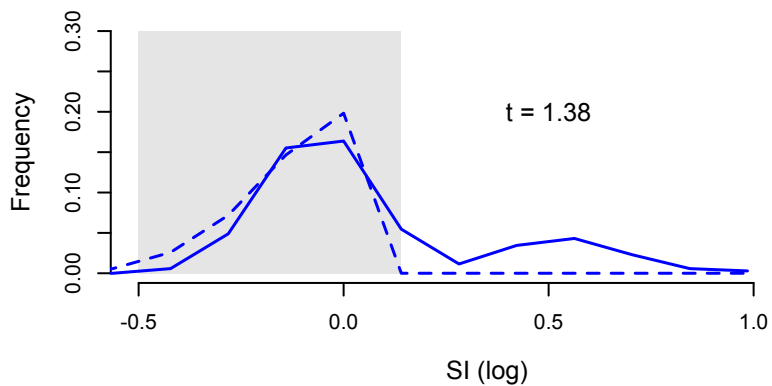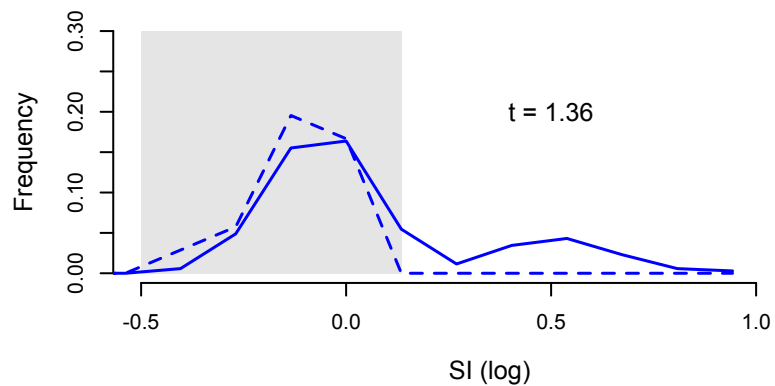**B**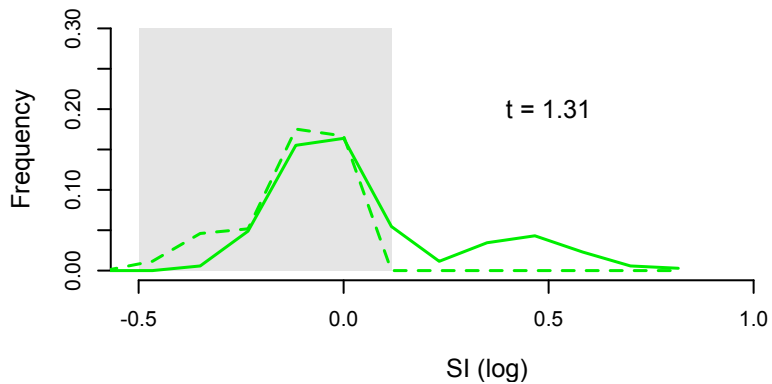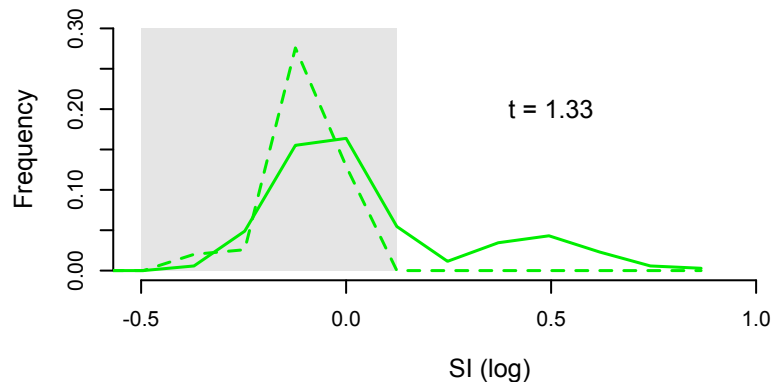**C**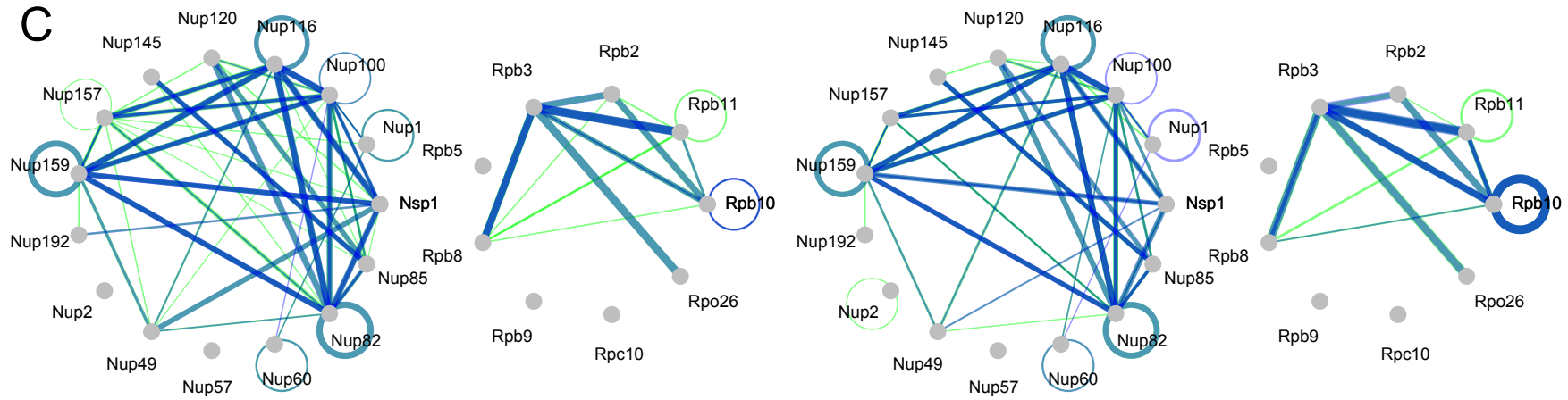

Supplement: Figure S9 — Comparison of SI values after correction by regression analysis between Skud and hybrid 1 (left) or hybrid 2 (right). (A) Estimation of the SI threshold value (t) for the detection of PPIs in Skud. (B) Estimation of the SI threshold value (t) for the detection of PPIs in hybrids. In each figure, distribution of SI values (log10) is shown with solid lines representing SI measured within complexes (SW values) and dotted lines representing SI measured among complexes (SA values). The t value corresponds to the maximal SA value measured in the diploid. Grey frames indicate background growth (SI<t). (C) Overlapped networks of Skud and hybrids Only SI above t and comparable interactions are represented. Line width is proportional to SI values measured between proteins in Skud (blue) and hybrids (green) in the NPC (left) and the RNApII (right). Interactions appear in turquoise when Skud and hybrid SI values overlap. Different degrees of turquoise depend in whether the interaction could be tested in reciprocal ways or not. (PDF) [file pgen.1003161.s011.pdf]

A

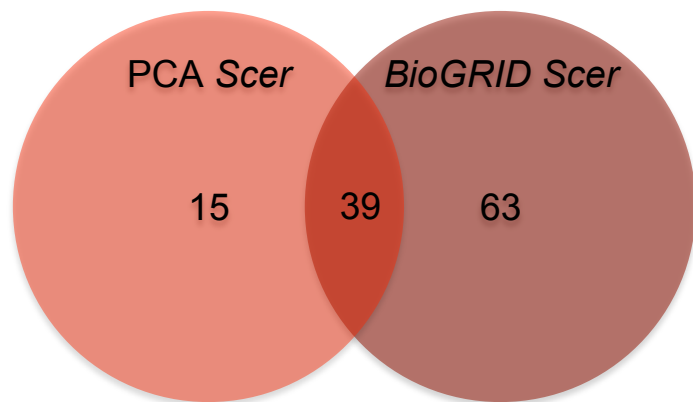

B

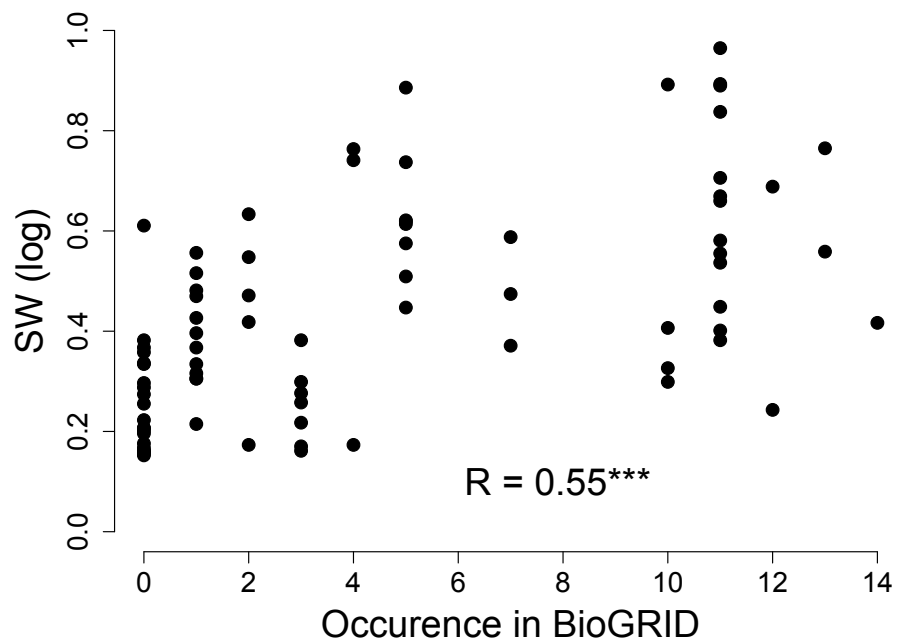

C

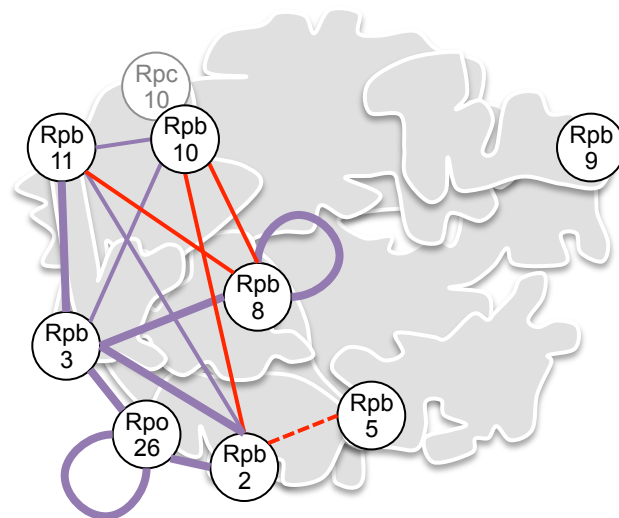

D

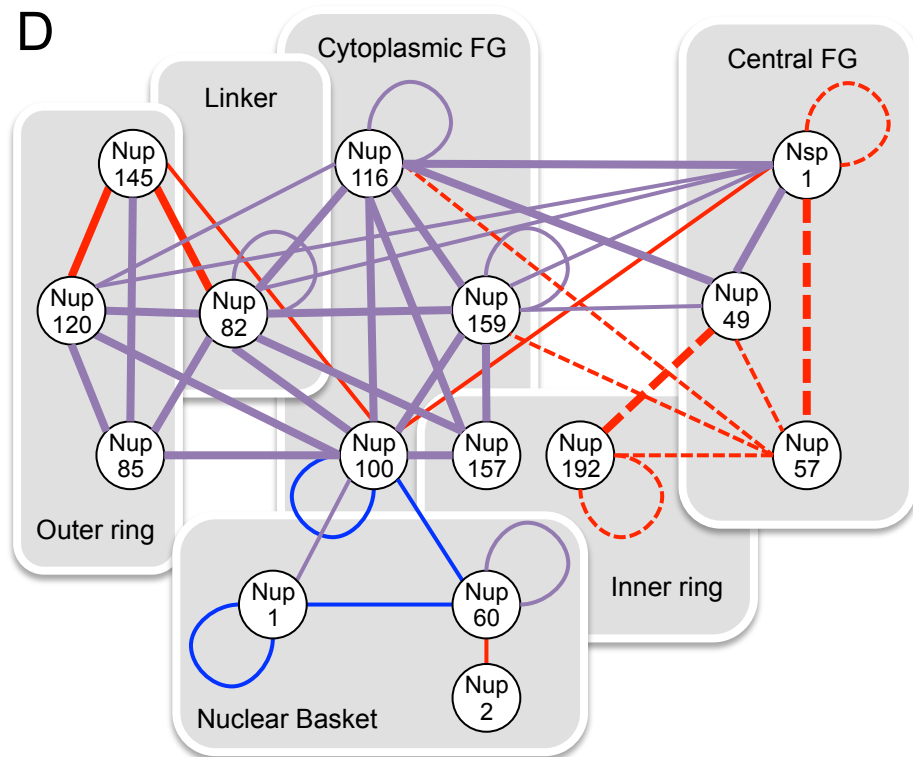

Supplement: Figure S10 — (A) Venn diagram showing the overlap of PPIs in Scer for NPC and RNApII identified by other physical methods than PCA (BioGRID). (B) Plot of 82 SW values corresponding to 54 PPIs we identified by PCA in Scer, against the number of occurrences of the PPI in BioGRID (Pearson's correlation; p<0.001). (C) Schematic representation of the RNApII complex based on crystal structure [35]. Protein names are located at the approximate position of the C-terminal of each protein. Lines indicate interactions detected only in Scer (red), only in Skud (blue) or in both species (purple). Dotted lines indicate interactions only detected in Scer because the information was not available in Skud. Bold lines indicate PPIs that were identified in reciprocal combinations. Only PPIs with SW>t are showed. The Rpc10 C-terminal is hidden by other proteins and Rpb9 is located at the opposite side of the complex, which was in agreement of the absence of PPIs for these proteins. (D) Schematic representation or the NPC organization based on structural data [34]. (PDF) [file pgen.1003161.s012.pdf]

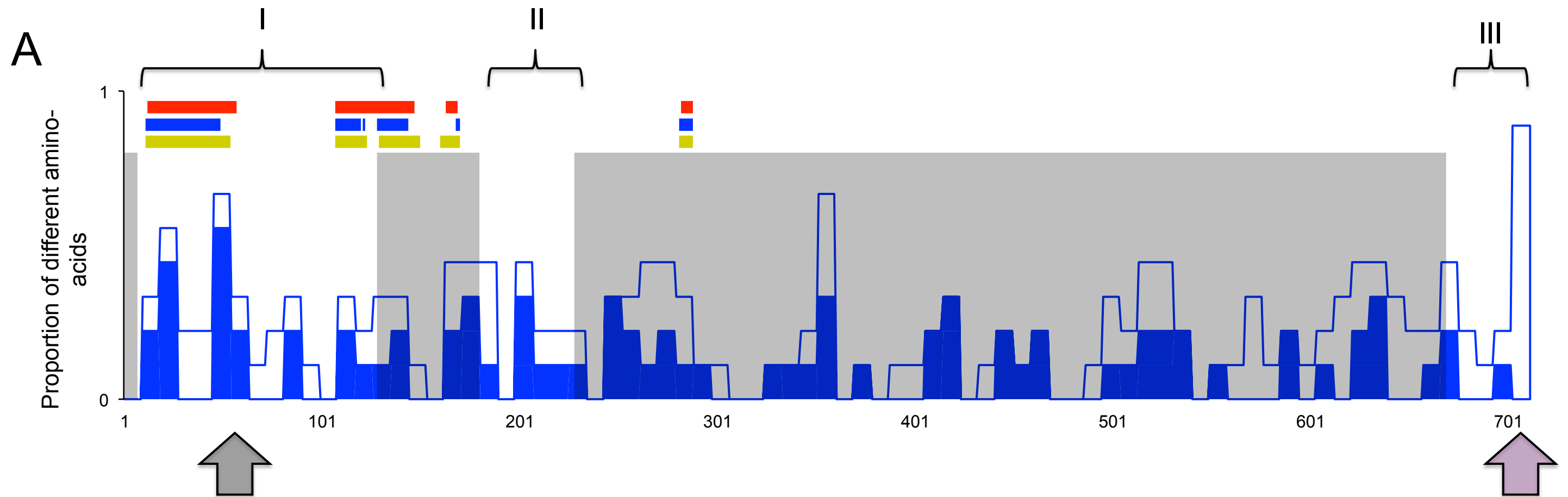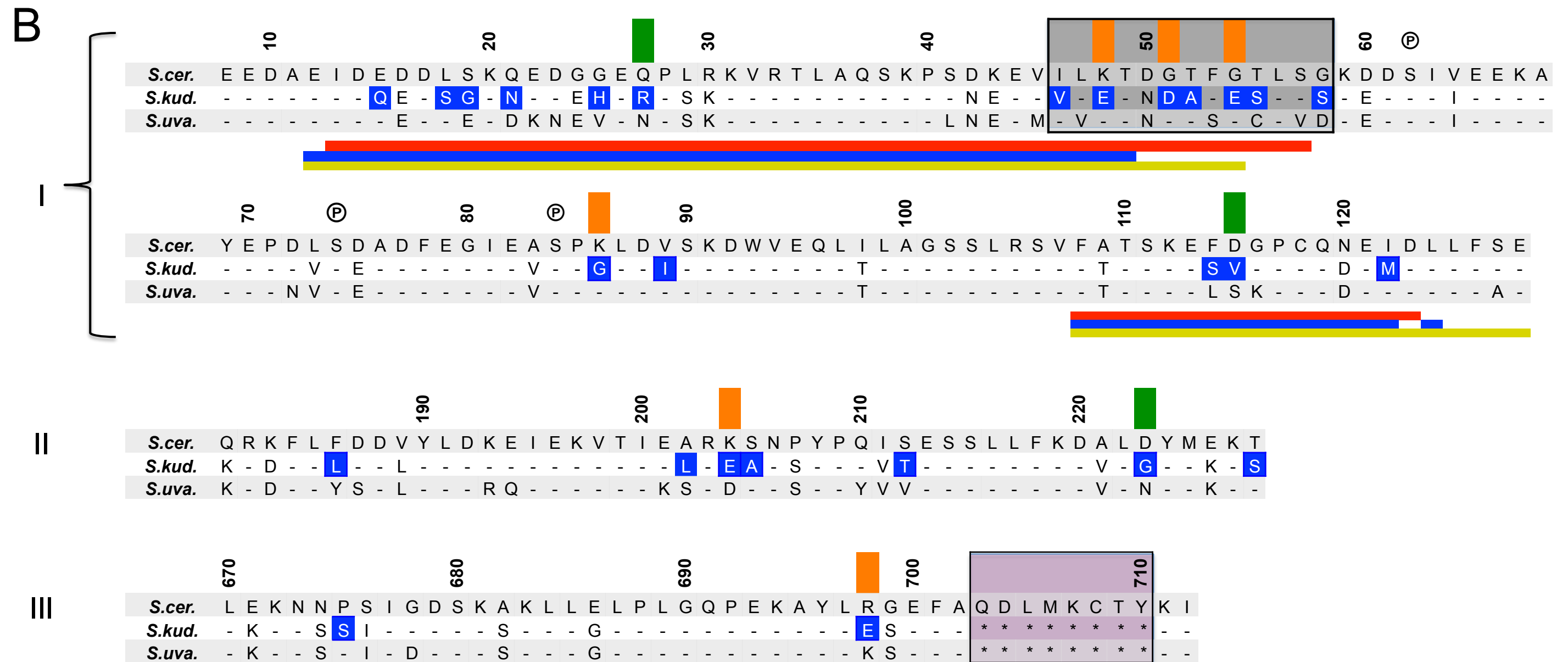

Supplement: Figure S11 — Amino acid (aa) divergence between Scer, Skud and Suva for the Nup145C protein. (A) Distribution of aa changes along the Nup145C protein (proportion of aa changes in a window of 9 aa). Changes filled in blue are unique to Skud. Domains I, II and III involved in the Nup145C-Nup120 interaction are indicated by brackets. Red, blue and yellow bars indicated disordered regions of the protein in respectively Scer, Skud and Suva, predicted by DISOPRED2 [63]. Grey and purple arrows indicate locations of respectively a high concentration of aa changes proper to Skud in domain I and a 8 aa insertion proper to Scer in domain III. (B) Detail of the aa divergence between three species in domains I, II and III with Scer as reference sequence. Dashes indicate no aa change; asterisks indicate deletions. Changes proper to Skud are indicated in blue. Green and orange positions indicate respectively positive and negative aa polarity changes in Skud. Phosphorylation sites are indicated by circled P. Grey and purple frames indicate locations of respectively the high concentration of aa changes specific to Skud in domain I and the 8 aa insertion proper to Scer in domain III. Predicted disordered regions are also indicated. (PDF) [file pgen.1003161.s013.pdf]
