# Supplementary material for: Evidence for the Robustness of Protein Complexes to Inter-Species Hybridization
Source: PLoS Genet. 2012 Dec 27;8(12):e1003161. doi: 10.1371/journal.pgen.1003161 (PMC3531474; doi:10.1371/journal.pgen.1003161)
Supplement: Figure S12 — Optimization of the DHFR-PCA in four Saccharomyces species. Difference in growth among positive (zipper-linker) and negative (linker) controls on methotrexate (MTX) tested in each species and each condition: MTX concentration (columns), incubation temperature and culture OD600 (rows). Each box represents a combination of conditions within a species (three replicates for each combination). The grey scale is proportional to the difference in relative spot growth (averaged among three replicates) between positive and negative controls (scale on bottom). In each condition, differences were tested using a t-test (***: p<0.001; **: p<0.01; *: p<0.05; n.s.: p>0.05). (PDF) [file pgen.1003161.s014.pdf]

T°	OD 600nm	**MTX 0.5%**				**MTX 1.0%**				**MTX 2.0%**			
		S. cer.	*S. par.*	*S. kud.*	*S. uva.*	*S. cer.*	*S. par.*	*S. kud.*	*S. uva.*	*S. cer.*	*S. par.*	*S. kud.*	*S. uva.*
**22°C**	0.0002	*	*	***	*	**	*	***	**	*	*	**	*
	0.002	**	**	***	*	**	**	***	*	*	***	***	*
	0.02	**	***	***	n.s.	**	***	***	*	**	*	**	**
	0.2	**	***	***	*	**	**	***	*	*	***	***	*
	2	*	*	*	n.s.	*	*	*	*	*	*	*	*
**25°C**	0.0002	***	*	**	***	*	*	***	***	***	*	**	*
	0.002	***	**	**	**	***	*	***	***	***	**	**	**
	0.02	*	***	***	*	***	***	***	**	**	**	**	***
	0.2	**	***	***	*	***	**	***	*	**	**	***	**
	2	*	*	***	*	**	**	***	*	*	*	*	*
**30°C**	0.0002	**	*	**	*	**	*	***	*	***	n.s.	***	*
	0.002	**	*	**	*	***	**	***	*	***	*	***	**
	0.02	***	**	***	**	***	***	***	**	***	**	***	***
	0.2	**	**	***	***	***	***	***	**	***	***	***	***
	2	*	*	*	**	**	**	**	**	*	**	*	*

0.0 0.8
