# Supplementary material for: Evidence for the Robustness of Protein Complexes to Inter-Species Hybridization
Source: PLoS Genet. 2012 Dec 27;8(12):e1003161. doi: 10.1371/journal.pgen.1003161 (PMC3531474; doi:10.1371/journal.pgen.1003161)
Supplement: Figure S13 — Design of the DFHR-PCA screen for the NPC and RNApII complexes. For each species and hybrids, MATa-DHFR[1,2] strains (top left) were incubated to saturation in 96-position pre-culture plates and divided in three parts (dotted lines), each containing one replicate of one of 24 MATa-DHFR[1,2] strains positioned at random. Cultures were printed four times with a 96-pin tool on a 86×128 mm plate (omnitray) filled with 35 ml of solid YPD medium with nourseothricin (100 mg/L), to obtain a 384-positions array. For each MATα-DHFR[3] strain (top right), an empty omnitray was filled with 20 ml of a fresh saturated liquid YPD culture and cells were transferred with a 96-position pin-tool on a plate with 35 ml of solid YPD and hygromycin B (250 mg/L). Four MATα strains were positioned per omnitray in order to obtain an interlaced array of 384 positions. 384-plates of MATa and MATα strains were crossed using a 384-pin tool on an omnitray with 35 ml of solid YPD. After incubation, colonies were transferred onto a 1536-array omnitray with 35 ml on solid YPD with nourseothricin and hygromycin B to allow diploid selection. Then, cells were transferred onto an omnitray filled with 35 ml of solid synthetic medium without adenine and with 2% methotrexate. (PDF) [file pgen.1003161.s015.pdf]

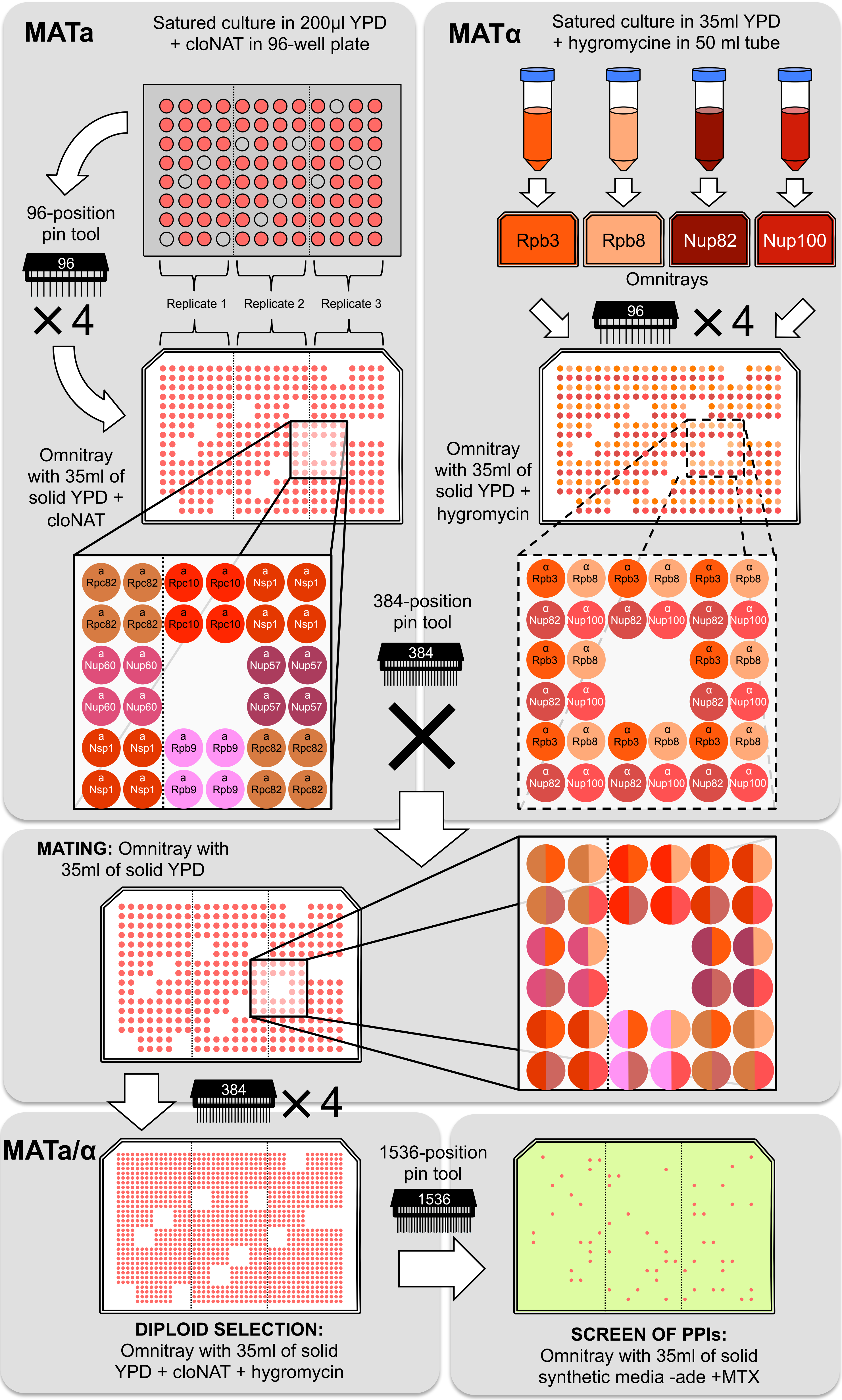

MATa
Satured culture in 200µl YPD + cloNAT in 96-well plate
96-position pin tool
96
× 4
Replicate 1
Replicate 2
Replicate 3
Omnitray with 35ml of solid YPD + cloNAT
a Rpc82 a Rpc82 a Rpc10 a Rpc10 a Nsp1 a Nsp1
a Rpc82 a Rpc82 a Rpc10 a Rpc10 a Nsp1 a Nsp1
a Nup60 a Nup60 a Nup57 a Nup57
a Nup60 a Nup60 a Nup57 a Nup57
a Nsp1 a Nsp1 a Rpb9 a Rpb9 a Rpc82 a Rpc82
a Nsp1 a Nsp1 a Rpb9 a Rpb9 a Rpc82 a Rpc82
MATα
Satured culture in 35ml YPD + hygromycine in 50 ml tube
Rpb3
Rpb8
Nup82
Nup100
Omnitrays
96
× 4
Omnitray with 35ml of solid YPD + hygromycin
α Rpb3 α Rpb8 α Rpb3 α Rpb8 α Rpb3 α Rpb8
α Nup82 α Nup100 α Nup82 α Nup100 α Nup82 α Nup100
α Rpb3 α Rpb8 α Rpb3 α Rpb8
α Nup82 α Nup100 α Nup82 α Nup100
α Rpb3 α Rpb8 α Rpb3 α Rpb8 α Rpb3 α Rpb8
α Nup82 α Nup100 α Nup82 α Nup100 α Nup82 α Nup100
384-position pin tool
384
MATING: Omnitray with 35ml of solid YPD
384
× 4
MATa/α
DIPLOID SELECTION: Omnitray with 35ml of solid YPD + cloNAT + hygromycin
1536-position pin tool
1536
SCREEN OF PPIs: Omnitray with 35ml of solid synthetic media -ade +MTX
