# Supplementary material for: Evidence for the Robustness of Protein Complexes to Inter-Species Hybridization
Source: PLoS Genet. 2012 Dec 27;8(12):e1003161. doi: 10.1371/journal.pgen.1003161 (PMC3531474; doi:10.1371/journal.pgen.1003161)
Supplement: Table S1 — List of DHFR-PCA strains used in this study. (DOCX) [file pgen.1003161.s016.docx]

| Complex | ORF | Gene | *S. cerevisiae* | | *S. kudriavzevii* | | *S. uvarum* | |
| --- | --- | --- | --- | --- | --- | --- | --- | --- |
|  |  |  | BY4741  (*MATa*) | BY4742  (*MATα*) | FM1109  (*MATa*) | FM1110  (*MATα*) | MG032  (*MATa*) | JBL033  (*MATα*) |
| NPC | YJL041W | *NSP1* | ‡ | ‡ | * | * |  |  |
|  | YOR098C | *NUP1* | ‡ | ‡ | * | * |  |  |
|  | YKL068W | *NUP100* | ‡ | ‡ | * | * |  |  |
|  | YMR047C | *NUP116* | ‡ | ‡ | * | * |  |  |
|  | YKL057C | *NUP120* | ‡ | ‡ | * | § | § | § |
|  | YGL092W | *NUP145* | ‡ | ‡ | * | * | § | § |
|  | YER105C | *NUP157* | ‡ | ‡ | * | * |  |  |
|  | YIL115C | *NUP159* | ‡ | * | * | * |  |  |
|  | YJL039C | *NUP192* | ‡ | ‡ | * | † |  |  |
|  | YLR335W | *NUP2* | * | ‡ | * | * |  |  |
|  | YGL172W | *NUP49* | ‡ | ‡ | * | † |  |  |
|  | YAR002W | *NUP60* | ‡ | ‡ | * | * |  |  |
|  | YJL061W | *NUP82* | ‡ | ‡ | * | * |  |  |
|  | YJR042W | *NUP85* | ‡ | ‡ | * | * | § | § |
|  | YGR119C | *NUP57* | ‡ | ‡ | † | † |  |  |
| RNApII | YOR210W | *RPB10* | * | * | * | * |  |  |
|  | YOL005C | *RPB11* | * | ‡ | * | * |  |  |
|  | YOR151C | *RPB2* | ‡ | ‡ | * | † |  |  |
|  | YIL021W | *RPB3* | ‡ | * | * | * |  |  |
|  | YBR154C | *RPB5* | ‡ | ‡ | † | † |  |  |
|  | YOR224C | *RPB8* | * | ‡ | * | * |  |  |
|  | YGL070C | *RPB9* | * | ‡ | * | * |  |  |
|  | YHR143W-A | *RPC10* | * | ‡ | * | † |  |  |
|  | YPR187W | *RPO26* | ‡ | * | * | † |  |  |
| * strains constructed in this study  † unsuccessful constructions  ‡ *Scer* strains already available from *Tarrassov et al.* (2008) and confirmed in this study  § *Suva* and *Skud* strains constructed for the dissection of Nup145-Nup120 interaction | | | | | | | | |
