# Supplementary material for: Evidence for the Robustness of Protein Complexes to Inter-Species Hybridization
Source: PLoS Genet. 2012 Dec 27;8(12):e1003161. doi: 10.1371/journal.pgen.1003161 (PMC3531474; doi:10.1371/journal.pgen.1003161)
Supplement: Table S2 — Summary of testable PPIs in Scer, Skud and their hybrids. Details of calculation are given under the table. Codes for different testable combinations of PPIs: S: homomeric interactions (P1-P1); NR: non-reciprocal interactions (only P1-P2 or P2-P1 is testable); R: reciprocal interactions (both P1-P2 and P2-P1 are testable). (DOCX) [file pgen.1003161.s017.docx]

| Crosses | Strains avalable | |  | Testable combinations | | | |  | Testable PPIs^5^ |
| --- | --- | --- | --- | --- | --- | --- | --- | --- | --- |
|  | *MATa* | *MATα* |  | All^1^ | S^2^ | NR^3^ | R^4^ |  |  |
| *S. cerevisiae* | 24 | 24 |  | 576 | 24 | 0 | 552 |  | 300 |
| *S. kudriavzevii* | 22 | 17 |  | 374 | 17 | 85 | 272 |  | 238 |
| Hybrid 1 (*Scer* *MATa* x *Skud* *MATα*) | 24 | 17 |  | 408 | 17 | 119 | 272 |  | 272 |
| Hybrid 2 (*Skud* *MATa* x *Scer* *MATα*) | 22 | 24 |  | 528 | 22 | 44 | 462 |  | 297 |
| (1) *MATa ×MATα*  (2) *MATa* ∩ *MATα*  (3) (*MATa* ∩ *MATα*) *×*(*MATa* – *MATα*)  (4) (*MATa* ∩ *MATα*)^2^ - (*MATa* ∩ *MATα*)  (5) S + NR + R/2 | | | | | | | | | |
