# Supplementary material for: Evidence for the Robustness of Protein Complexes to Inter-Species Hybridization
Source: PLoS Genet. 2012 Dec 27;8(12):e1003161. doi: 10.1371/journal.pgen.1003161 (PMC3531474; doi:10.1371/journal.pgen.1003161)
Supplement: Table S3 — Original strains used in this study. (DOCX) [file pgen.1003161.s018.docx]

| Species  (Original strain) | Specific temperature | | Strain | Genotype | Reference |
| --- | --- | --- | --- | --- | --- |
|  | Incubation | Heat-shock |  |  |  |
| *S. cerevisiae*  (S288c) | 30°C | 42°C | BY4741* | *MATa Δho* ‡§\|\|**** | [[1](#_ENREF_1)] |
|  |  |  | BY4742* | *MATα Δho* †‡§\|\| |  |
| *S. paradoxus*  (CBS432) | 30°C | 37°C | JRY9134 | *MATα ho::NAT* †‡ | constructed as described in [[2](#_ENREF_2)] |
|  |  |  | MG030* | *MATα ho::URA3* †‡ | This study |
|  |  |  | JBL026* | *MATa Δho* †‡ | This study |
| *S. kudriavzevii*  (ZP591) | 22°C | 34°C | FM1109* | *MATa ho::KanMX4* | [[3](#_ENREF_3)] |
|  |  |  | FM1110* | *MATα ho::KanMX4* |  |
| *S. kudriavzevii*  (IFO1802) | 25°C | 37°C | GIL2022* | *MATa ho::KanMX4* | [www.genomics.princeton.edu/glang/](http://www.genomics.princeton.edu/glang/) |
|  |  |  | GIL2023* | *MATα ho::KanMX4* |  |
| *S. uvarum*  (CBS7001) | 25°C | 37°C | JRY8153 | *MATa ho::NAT* †‡§¶ | [[2](#_ENREF_2)] |
|  |  |  | MG032* | *MATa ho::URA3* †‡§¶ | This study |
|  |  |  | JBL033* | *MATα Δho* †‡§¶ | This study |
| *Strains used for PCA in this study  Auxotrophies: † *lys2,* ‡ *ura3,* § *his3,* ¶ *trp,* \|\| *leu2, **met15* | | | | | |

1. Brachmann CB, Davies A, Cost GJ, Caputo E, Li J, et al. (1998) Designer deletion strains derived from Saccharomyces cerevisiae S288C: a useful set of strains and plasmids for PCR-mediated gene disruption and other applications. Yeast 14: 115-132.

2. Gallagher JE, Babiarz JE, Teytelman L, Wolfe KH, Rine J (2009) Elaboration, diversification and regulation of the Sir1 family of silencing proteins in Saccharomyces. Genetics 181: 1477-1491.

3. Hittinger CT, Goncalves P, Sampaio JP, Dover J, Johnston M, et al. (2010) Remarkably ancient balanced polymorphisms in a multi-locus gene network. Nature 464: 54-58.
