# Supplementary material for: Evidence for the Robustness of Protein Complexes to Inter-Species Hybridization
Source: PLoS Genet. 2012 Dec 27;8(12):e1003161. doi: 10.1371/journal.pgen.1003161 (PMC3531474; doi:10.1371/journal.pgen.1003161)
Supplement: Table S4 — List of oligonucleotides used in this study. (DOCX) [file pgen.1003161.s019.docx]

| Primer name (species) | Sequence |
| --- | --- |
| URA3 *cassette amplification* |  |
| Spar-HO-5 (*Spar*) | CACACCCTTATAAGCAGCAATCAATTTCATCTAACTTCAACCAGCTGAAGCTTCGTACGC |
| Suva-HO-5 (*Suva*) | CATCTATATAGACAGCAATCACTTCCATCTAGCCTTTAAACCAGCTGAAGCTTCGTACGC |
| Spar-HO-3 (*Spar*) | TTATCACATACAACTTTTTTTTAACTAATGTACACATTGCATAGGCCACTAGTGGATCTG |
| Suva-HO-3 (*Suva*) | TACATACAACCTACTTTTTTTAAACTAATTTACATATTGCATAGGCCACTAGTGGATCTG |
| *Locus* HO *specific primers* | |
| O3-1 (*Spar*) | ACAGAAGCTTGTTGAAGTGC |
| O3-2 (*Spar*) | CATTCAAGTAAAGAGATCACC |
| O3-3 (*Suva*) | AGCCAATTCATACTCTTAGCC |
| O3-4 (*Suva*) | TCGATCAATGGAGTGACCG |
| *Mating-type verification* |  |
| verif*MATa/α*-R-Suva (*Suva*) | AGTCACATCAAGATCATTTATGG |
| verif*MAT*a/α-R-Spar (*Spar*) | AGTCACATCAAGGTCATTTATGG |
| verif*MATa*-F-Suva (*Suva*) | ACGCCACTTCAAGTAAGAGTCTG |
| verif*MATa*-F-Spar (*Spar*) | ACTCCACTTCAAGTTAGAGTTTG |
| Verif*MATα*-F (*Spar/Suva*) | GCACGGAATATGGGACTACTTCG |
| *Construction of* p-41-ZL-DHFR[1,2] *and* p-41-ZL-DHFR[3] *plasmids* | |
| Zipper_linker_F | GCCGCTCTAGAGGGATG |
| Zipper_linker_R | GTCAGGATCCAGAACCTCCA |
| DHFR1,2_NAT_F | AACATCGGATCCATGGTTCGACCATTGAACTGC |
| AfeI_TEFterm_R | TATTAGCGCTTCGACACTGG |
| DHFR3_HPH_F | ATCGGCGGATCCATGAGTAAAGTAGACATGGTTTGGATAG |
| *Reverse primer for the genotyping of DHFR strains (See Table S4 for forward primers)* | |
| ADHTerm-R | CCATCTTTTCGTAAATTTCTG |
| Construction of *Scer* mutant for and *SkNup145* | |
| OP37-A1 (*SkNup145*) | TTTTTAGAACCACAAGCAAAGGAGAAGCAGTAGCCACCTTATGTTCAACAAAAGCGTAAA |
| OP37-C3 (*SkNup145*) | TGGCGAAGAAGTCCAAAGCTTTAAATTTTGGCAAACTCGC |
| OP37-D3 (*ADH-NATMX*) | GCGAGTTTGCCAAAATTTAAAGCTTTGGACTTCTTCGCCA |
| OP37-B1 (*ADH-NATMX*) | TTACTATTTTTCTTTTTTTTAGAAATAAAAATAAAAAAACTTCGACACTGGATGGCGGCGTTAG |
| OP37-E01 | TCAGTGGAGTTCTTCCTTCATTTAC |
| OP37-H01 | ATTTGGCACGGTAGATCCTG |
| OP32-E08 | AGGATCTACCGTGCCAAATG |
| OP37-E02 | CTCACTTGGCATACGCTGAA |
| OP37-D02 | TGAAACAAATGACGCTTTCG |
