# Supplementary material for: Evidence for the Robustness of Protein Complexes to Inter-Species Hybridization
Source: PLoS Genet. 2012 Dec 27;8(12):e1003161. doi: 10.1371/journal.pgen.1003161 (PMC3531474; doi:10.1371/journal.pgen.1003161)
Supplement: Table S5 — List of oligonucleotides used for the DHFR-PCA strain construction. (DOCX) [file pgen.1003161.s020.docx]

| *Species* | *Gene* | Primers for cassette construction | | Forward primer for confirmation |
| --- | --- | --- | --- | --- |
|  |  | Forward* | Reverse† |  |
| *S. cerevisiae* | | | | |
|  | *NSP1* | ‡ | ‡ | GATGTTGTGTCTACAAGTTCAGGTG |
|  | *NUP1* | ‡ | ‡ | TGGTGCGAATGCTGCCTCTG |
|  | *NUP100* | ‡ | ‡ | AGTCATTGGTCACAGAAATTATGGT |
|  | *NUP116* | ‡ | ‡ | ACGCTTAAAGAAAAATCCAAATTCT |
|  | *NUP120* | ‡ | ‡ | AGAACTGCAAGACTTCAAAGAAAAA |
|  | *NUP145* | ‡ | ‡ | GGCAATTACCTTTCTGAAGTACAAA |
|  | *NUP157* | ‡ | ‡ | GGATTGACGAAGATTATAGAAAGCA |
|  | *NUP159* | GCAAATTGGTGATTTCTTCAAAAATTTGAACATGGCAAAA- | TTATTAACGGCACTAACAACGTACATATAGCTAAATATCA- | CTAACATTCAAGACCTGAAGGAAAA |
|  | *NUP192* | ‡ | ‡ | AATCCGCTACTAGTTGACAAAGATG |
|  | *NUP2* | ATTTACGAAAGCTATTGAAGATGCTAAAAAAGAAATGAAA- | AGGGTTCTATTCTATTTAAAATTGTTAACTGTATTTACTC- | TTGTAGACTCCTTCAAATATGAGCC |
|  | *NUP49* | ‡ | ‡ | TACTGAAGTCAGAATCTGCAACAAG |
|  | *NUP57* | ‡ | ‡ | AAATTAATGGTTTTCAACGACGATA |
|  | *NUP60* | ‡ | ‡ | ATTTCAATGTTCCCGTGGTGTCTA |
|  | *NUP82* | ‡ | ‡ | GAAAATTCGAAGCACAAAATAAGAA |
|  | *NUP85* | ‡ | ‡ | TTGAATTCCCTTATTTACCAAAACA |
|  | *RPB10* | TGAAAAGTTTTTAAGATACAACCCATTAGAAAAAAGAGAT- | AGGAAAAGAAAAGTTCAAAAACAGCCATATTGAGGAACAC- | AAGGTACTGCATTGTCAAGATTAGG |
|  | *RPB11* | CGAGTGGAACCTGCAAACTCTAGCCGCCGACGACGCATTT- | CTGTTTAGATTTTATGGCTATAGTATTAAACTCCTATATA- | AGAACTTCTGAACGACAGAAAAGTG |
|  | *RPB2* | ‡ | ‡ | AGGTATATCCAAACTACTGCGTGAG |
|  | *RPB3* | AATGGGTAATACTGGATCAGGAGGGTATGATAATGCTTGG- | GTTCACTTGTTTTTTTTCCTCTATTACGCCCACTTGAGAA- | ATCTCAAATGGGTAATACTGGATCA |
|  | *RPB5* | ‡ | ‡ | AAGGACAGGGGTTATTTTATCACTC |
|  | *RPB8* | TAACTTGAAGCAAGAGAACGCTTATCTTTTGATTCGTCGT- | TAAGTGATCGCCCTTTTTTTTAGCACTCGGTCCAATGCTG- | GGATCTAATTGCCGTTTACTACTCA |
|  | *RPB9* | ATTTACTTCAGATCAAAAAAACAAAAGGACGCAGTTTTCA- | TCTCTCCCTCTGTCATTAATTTTGAAAGTTCGTTGAGCAC- | GAGATCTGATAGAGAATGTCCCAAA |
|  | *RPC10* | GAAGGCTAGGACTAAGAGATTGGTTCAATTTGAAGCTAGA- | TTCTGCCCCGGTAACCCATTATGGTTTTCAGTCTCTCCAT- | GGCAACTTTGAAGTATATTTGTGCT |
|  | *RPO26* | CTTTGAGGACTGGAGTGTGGAGGAACTCATTGTGGATTTG- | CAAACAAAGCAAATACACATAAAGGAAAATGCAGGTGTAA- | AAGAGCAACTACTCCATACATGACC |
| *S. kudriavzevii* (ZP591) | | | | |
|  | *NSP1* | CTCCTCTTCTTTGGAAAAAAAGATTAGTTCGATAAAGAAA- | CGTATGTTCGATTAGGTCAAAAAGAAAATCTTTTTTTCAA- | CGGAGATCAACAACGTCAGT |
|  | *NUP1* | GGCAAACAGAAAAATTGCAAGAATGAGGCATTCCAAAAGG- | AAAAAGAAACGGTACAATACTTATTAATATACCCCTTTAA- | CAGGTGGGACACCTTCATTT |
|  | *NUP100* | AGGTACTTATAGTTATACTGTAGACTCACCAGTTTTACCT- | AGACTACTAAAACAGGACAAGTGTTCCTAAAAAAATATAA- | AGCTATGACCCCGTAACAGG |
|  | *NUP116* | TTACGTGTTTATCGTAAACTATGCAGCACAGCATTCCTTT- | ATTTAACTCTCTCTTCTATATAGTTTGATTATATCTTTAT- | TTTGAAAGTTATGATGCCGC |
|  | *NUP120* | GGTAACTTTAACTGACTTGAGGGACGAATTGAGGAATCTA- | AAAATGAGGAGTACTAATTTACACTTGTATAAACGATTGC- | CTTGAACAGTAATAGAGTGG |
|  | *NUP145* | GCCTGAAAAGGCTTACTTGGAGAGCGAGTTTGCCAAAATT- | AAGATAAAAGATAATTTACATGTCTTATATGTACATTTCA- | CGTGAAGTTCCTGCATGTTG |
|  | *NUP159* | GCAAATCGGTGATTTTTTCAAGAGTCTAAACCTGGCGAAA- | TATTAATGGCGTTGGACCGTAGATATATCTACATAGTCTA- | AATACGAAGAAGCAAATCGG |
|  | *NUP192* | TGTAATGCTGTGCACTTTAACAGGATACCAAAACGAGGAC- | CTGCAAAATTCATTAGTCCCCTACTCACAAGTAAGACGAC- | GGCTAATTGTAATGCTGTGC |
|  | *NUP2* | CATGAAAGCGATCGAAGATGCTAAAAAAGAAATGAAAAAT- | TAAAGATTCTGCTTTATGTAAATTTGTTTTTTTGTATTTA- | AACAGAAGGAAGAAGGTCGC |
|  | *NUP49* | TCTACACCAAAAAATGAAATCATTGGCATCAAAAACCATA- | GTACTTATTTTACGAAGTATATAACCGTCAGAACAGGAAC- | GAGGATCGCTGTTCTACACC |
|  | *NUP57* | AAAGGATGCCGCAATGGTGAAAGAGTATACAAATAAGACG- | ATCCTATGAATTAATATATTCACTGAATTAAACATCCTAA- | GTTTTGGAAAAGGATGCCGC |
|  | *NUP60* | TGAAAACAAAGTTGAGGCCTTCAAATCTTTGTATACTTTT- | GGGTTATACAGTAATTATGGCACGGTGAGAACTCTCACCA- | TCCGTACCAAAACGACAACA |
|  | *NUP82* | TGTTTCCCAGCAACTCACCACTAATACTCAGTCAAAAAAC- | TAGTCTTTGCCATACAAATATTGATAACAGCCGGTGCCAT- | TGCGCAAGATGCTAGAAGAA |
|  | *NUP85* | AAGAAAAAAGTTGAATTTCAAGTTATGTCAAGCCTTCATG- | TTCCTAAAATACTATCTCAAAAAAAAACCACTGTACCGAT- | ACCTGATAGTACAGCGGAGC |
|  | *RPB10* | TGAGAAGTTTCTAAGATATAACCCACTGGAAAAGAGGGAT- | AAGGAAAGAGTCTAAAAGAAAAAGCTGTGATAATAAACAT- | GGTACCGCGTTGTCAAGATT |
|  | *RPB11* | GAACCTACAGACTTTGGCCGCCGACGATGCATTCAATATG- | CTCTGTTGCTGTCTAAATTTCAGCGTCTGGTATTAAATGC- | CTTCGAGACAGAGTGGAACC |
|  | *RPB2* | CATAACACCACGTTTATATACGGATCGTTCAAGAGATTTT- | TTTTCAGGTTTTATTTTCTTTAAAGTTACAACATTATTTT- | ACGGTCATTGCCAAATTGA |
|  | *RPB3* | AATGGGTAATACTGGATCAAGTGGGTACGACAATGCTTGG- | GCTTGATTGTTTGCCCCTCTTATACACCCACACTCAGAGA- | CAAGATCCTTATTCCAATGC |
|  | *RPB5* | TGAAACCTCGGGTCGTTATGCCAGTTACAGGATCTGTATG- | TTTGCTATCGTTTGTAAAGAAGTAGAACCAAGCTGTCGAT- | AGAAAGAGTGAAACCTCGGG |
|  | *RPB8* | TAACTTGAAGCAAGAAAATGCTTATCTTTTGATTCGTCGT- | TATCAGTAAGTGATCGTTTCCTCTTTTCAGCTTAACGCTG- | CGTTTACTACTCATTCGGCG |
|  | *RPB9* | ATTTACTTCTGATCAAAAGAATAAAAGAACGCAGTTTTCG- | TTTCTCCCCTTTTGATTAGTTTTCACAAGTTGTCTGACGC- | CGTGTGTTTGTCTTGCTCGC |
|  | *RPC10* | GAAAGCTAGAACTAAGAGATTGGTTCAATTCGAAGCTAGA- | GCCCTGCCAATTCCATGCATGGCTCTTAGCTTTTCTTACC- | GTCTCGTGAAGGGTTCCAAA |
|  | *RPO26* | CTTCGAAGATTGGAGTGTGGAGGAACTGATTGTGGATTTG- | AACAAAACAAATACACATATGGAAAAAAATGCAGATGCAA- | CATTGCGTATTGCCATGAAG |
| *S. uvarum* (CBS7001) | | | | |
|  | *NUP120* | Gataacattaaccgatataaaagacgaactaagcaatatc- | Aacaaataaagtactaatttacagttacataaattatttg- | GCAGAGTGATAACATTAACC |
|  | *NUP145* | ACCCGAAAAGGCATACTTAAAGAGCGAATTTGCCAAAATT- | AAAGATAAAATAATTTACTTCTTTCTTATATGTACACTTC- | ACATCGGAAACGATCCACTC |
|  | *NUP85* | Aagaaaaaaattgaattttaagttatgtcaaacgttcatg- | Tattatatgatataatacttcaaaacattaccgtaccagt- | TCTACCTGATAGTACATTGG |
| * the following sequence was added to each forward primer: -GGCGGTGGCGGATCAGGAGGC  † the following sequence was added to each reverse primer: -TTCGACACTGGATGGCGGCGTTAG  ‡ not used in this study | | | | |
