# Supplementary material for: Evidence for the Robustness of Protein Complexes to Inter-Species Hybridization
Source: PLoS Genet. 2012 Dec 27;8(12):e1003161. doi: 10.1371/journal.pgen.1003161 (PMC3531474; doi:10.1371/journal.pgen.1003161)
Supplement: Text S1 — Supplementary methods. (DOCX) [file pgen.1003161.s021.docx]

**Evidence for the Robustness of Protein Complexes to Inter-Species Hybridization**

Jean-Baptiste Leducq, Guillaume Charron, Guillaume Diss, Isabelle Gagnon-Arsenault, Alexandre K Dubé, & Christian R Landry

**Supporting Information**

# 1. Construction of DHFR-PCA compatible *S. paradoxus* (*Spar*) and *S. uvarum* (*Suva*) strains. We replaced the selectable marker *NatMX4* (resistance to nourseothricin) at the *HO* locus with *URA3* in strains JRY9134 and JRY8153 (Table S3). The *URA3* cassette (from *Klyveromyces lactis*) was amplified by PCR from *pUG72* [[1](#_ENREF_1)] using primers *Spar*-HO-5, *Spar*-HO-3, *Suva*-HO-5 and *Suva*-HO-3 (Table S4) and the high-fidelity polymerase Accuprime Pfx following manufacturer instructions (Invitrogen). Transformations were performed as in Tarassov *et al*. [[2](#_ENREF_2)] with the following modifications: heat shock was performed for 20-30' after adding 5 μl of Dimethyl sulfoxide (DMSO) and followed by a recovery in YPD (yeast extract, peptone, dextrose) for five to six hours (See Table S3 for specific temperatures). The reduction of DMSO volumes used here, as compared to 15 μl usually used for *Scer*, increased transformation efficiency by about 2-3 times in *Spar* and *Suva*. Cells were plated onto selective synthetic solid medium without uracil and incubated for three days at specific temperatures (Table S3). Correct replacement of the *NatMX4* cassette by the *URA3* gene was verified by colony PCR using primers URA-C [[1](#_ENREF_1)] along with primers O3-1 for *Spar* and O3-3 for *Suva* (Table S4) and URA-B [[1](#_ENREF_1)] along with O3-2 for *Spar* and O3-4 for *Suva* (Table S4). Colony PCRs were adapted from Huxley *et al*. [[3](#_ENREF_3)]: a small amount of fresh colonies was resuspended in 50 μl of NaOH 20mM and incubated for 30' at 95°C for cell lysis. Cells were centrifuged for 5' at 4000 rpm. For each PCR reaction, the mixture contained 2 μl of 10X Bioshop® Buffer, 2 μl of supernatant of the lysed cells, 1.2 μl of MgCl_2_ 25mM, 1.6 μl of dNTP mix 2.5 mM, 1.6 μl of each primer at 2.5 μM and 0.12 μl of Taq Polymerase Bioshop® 5 U/μl, in a final volume of 20 μl. PCR reactions were carried out in a thermocycler MasterCycler ProS Eppendorf© with the following steps: 5' at 94°C; then 35 cycles of 30” at 94°C, 30” at 57°C and 1' at 72°C; and a final extension of 3' at 72°C. PCR products were then size-verified on agarose gels. Cassette-replaced JRY9134 and JRY8153 were respectively renamed MG030 and MG032 (Table S3).

# We obtained isogenic strains with the complementary mating types by mating type switching. We first removed the *URA3* gene from the *HO* locus as follows. We transformed strains MG030 and MG032 with the plasmid *pNATCre* [[4](#_ENREF_4)] to induce the recombination of *Lox* regions flanking the *URA3* cassette. Transformations were carried out as described above with 600 ng of plasmid. Cells were incubated for two hours at a specific temperature (Table S3) before being plated onto solid YPD with nourseothricin (100 mg/L) and incubated for three days at a specific temperature (Table S3). One colony per strain was isolated and incubated in 5 ml of liquid YPD for 48 hours at 25°C with agitation to induce the expression of the recombinase. Then, 50 μl of culture (OD_600_ adjusted at 0.002 – 0.0002) were plated on complete synthetic solid medium with 5-FOA (1 g/L) and uracil (50 mg/L) to verify the loss of the *URA3* cassette. Plates were incubated for three days at 25°C and then replica-plated on solid YPD with nourseothricin (100 mg/L) to ensure that the *pNATCre* plasmid was lost. For each strain, one colony with negative growth on YPD/nourseothricin was isolated from the plate with 5-FOA. We then switched their mating types by transforming with the plasmid *pGAL-HO* (provided by M.C. Keogh) according to a high-efficiency transformation protocol [[5](#_ENREF_5)]. We adapted this method for *Spar* and *Suva,* which required a heat-shock at 37°C for optimal transformation efficiency. Then, cells were plated on selective solid synthetic medium without uracil and incubated at a specific temperature (Table S3) for three days. One colony was isolated for each species and incubated in 5 ml of liquid synthetic medium without uracil and with 0.1% of glucose and 2% of raffinose. After two days of incubation with shaking at room temperature, 1 ml of culture was added to 5 ml of liquid synthetic medium without uracil and with 0.1% of glucose and 2% of galactose and agitated three hours at 25°C to induce the expression of *HO* and the mating-type switching. Then, 5-10 μl of culture was plated on solid YPD and incubated for three days at 25°C. Mating-type switching was verified by colony PCR (see above) using primers described in table S4. The reverse primers were common to both mating types but species-specific. The mating-type switching of *Spar* strain MG030 and the *Suva* strain MG032 respectively provided strains JBL026 and JBL033 (Table S3).

**2. Construction of *p41-ZL-DHFR[1,2]* and *p41-ZL-DHFR[3]* plasmids**. DNA fragments containing the leucine zipper and the linker were amplified from *p413-ZL-VenusF1* [[6](#_ENREF_6)] with primers Zipper_linker_F and Zipper_linker_R (Table S4), including the *XbaI* site at the 5’ end of the amplicon and adding the *BamHI* site to the reverse primer at the 3’ end. This amplicon was then doubly digested and inserted between the *XbaI* and *BamHI* site of *p413-L-RLucF1* plasmid [[7](#_ENREF_7)], resulting in *p413-ZLL-RLucF1*. The *DHFR[1,2]* and *DHFR[3]* fragments associated with, respectively, the *NATMX* and *HPH* (resistance to hygromycin B) cassettes, were amplified from *pAG25-L-DHFR[1,2]* (with primers DHFR1,2_NAT_F and AfeI_TEFterm_R; Table S4) and *pAG32-L-DHFR[3]* (with primers DHFR3_HPH_F and AfeI_TEFterm_R; Table S4), adding an *AfeI* site at the 3’ end of both PCA fragments, and a *BamHI* site at the 5’end. These two PCR products were inserted in parallel between the *BamHI* and *AfeI* sites of *p413-ZLL-RLucF1*, replacing the *BamHI-L-RLucF1-HIS3-AfeI* and resulting in *p41-ZL-DHFR[1,2]* and *p41-ZL-DHFR[3]*. In each resulting plasmid, the *DHFR* fragment was fused downstream of the coding sequence of homodimerizing residues of the *GCN4* parallel coiled-coil leucine zipper (Z) via a sequence coding for a 15-amino-acid flexible linker (L) peptide [[8](#_ENREF_8)]. Centromeric plasmids *p41-ZL-DHFR[1,2]* and *p41-ZL-DHFR[3]* also contained respectively the *NatMX4* and the *HPH* resistance gene.

**3. Construction of DHFR-PCA haploid strains in *S. cerevisiae, S. paradoxus, S. kudriavzevii* and *S. uvarum***. Haploid strains of the four species were transformed as described above with plasmids *p41-ZL-DHFR[1,2]* or *p41-Linker-DHFR[1,2]* (*MATa*) and *p41-ZL-DHFR[3]* or *p41-Linker-DHFR[3]* (*MATα*). Temperatures for the heat-shock and incubation varied among species (See Table S3). After heat-shock, cells were directly plated onto selective medium. Crosses between haploid *MATa* and *MATα* strains were performed on plate with solid YPD medium by mixing strains and incubating them for two days at 25°C. Diploid selection was performed on solid YPD with nourseothricin 100 mg/L and hygromycin B 250 mg/L and incubated for two days at 25°C. A second diploid selection was performed. Diploid cells were transferred in 3 ml of liquid synthetic medium without adenine (see below for details). Cells were incubated with agitation at 25°C until OD_600_ = 2. For each species and each control (negative and positive), we tested the following conditions of growth on solid medium (2.5% noble agar): concentration of MTX (50, 100 and 200 mg/L), incubation temperature (22, 25 and 30°C) and cell concentration (OD_600_ = 2, 0.2, 0.02, 0.002 and 0.0002). The same experiment was performed in DMSO as control (MTX solvent). For one liter of solid medium, 6.69 g of yeast nitrogen base without amino acids and ammonium sulfate and 25 g of noble agar were separately sterilized 15 minutes at 121°C and then incubated for 30 minutes at 55°C. Yeast nitrogen base and noble agar were then combined, and sterile liquid glucose (final concentration 20 g/L) was added. A solution of amino acids that was previously sterilized by micro-filtration (0.2 μm filters) was also added in the medium after sterilization. One liter of medium contained 0.04 g of L-Trytptophane, 0.03 g of L-Tyrosine, 0.05 g of L-Phenylalanine, 0.1 g of L-Glutamic acid (monosodiumsalt), 0.1 g of L-Asparagine, 0.15 g of L-Valine, 0.2 g of L-Treonine, 0.375 g of L-Serine, 0.02 g of Uracile, 0.02 g of L-Histidine HCl, 0.02 g of L-Arginine HCl, 0.02 g of L-Methionine, 0.02 g of L-Lysine and 0.06 g of L-Leucine per litter. For PCA screen of PPIs, 20 ml of methotrexate dissolved in DMSO (10 mg/ml) or 20 ml of DMSO (for control) was added to the medium.

**4. Construction of DHFR-PCA haploid strains for the study of PPIs in the NPC and RNApII in *S. cerevisiae* and *S. kudriavzevii***. We constructed DHFR haploid strain of *Skud* for nine RNApII and 15 NPC proteins that showed at least one interaction within *Scer* in Tarrassov *et al.* [[2](#_ENREF_2)]. Cassettes containing *DHFR* gene fragments were amplified by PCR from plasmids *pAG25-L-DHFR[1,2]* and *pAG32-L-DHFR[3]* [[2](#_ENREF_2)]. Plasmid *pAG25-L-DHFR[1,2]* contained a construction consisting of the 15-amino-acid flexible linker peptide followed by a sequence coding for the *DHFR[1,2]* fragment and by the *NAT* gene controlled by the *TEF* promoter. Plasmid *pAG32-L-DHFR[3]* contained a construction constituted of a sequence coding for the 15-amino-acid flexible linker peptide followed by a sequence coding for the *DHFR[3]* fragment and by the *HPH* gene controlled by the *TEF* promoter. Oligonucleotides were designed as described in Tarrassov *et al.* [[2](#_ENREF_2)] (see Table S5 for a list of primers used for cassette amplification). Primers were designed from whole genome annotations and assemblies available for *Scer* and *Skud* [[9](#_ENREF_9)]. PCR amplifications were performed as follows. Reaction mixture contained 5-20 ng of either *pAG25-L-DHFR[1,2]* or *pAG32-L-DHFR[3]*, 4 μl of 5X KAPA HiFi™ Fidelity Buffer (KapaBiosystems), 0.6 μl of dNTP mix 10 mM, 0.4 U of KAPA Hifi™ (HotStart) DNA Polymerase and 0.6 μl of each primer at 10 mM, in a final volume of 20μl. PCR reactions were carried out in a thermocycler MasterCycler ProS Eppendorf© with the following steps: 5' at 95°C; then 32 cycles of 20'' at 98°C, 15'' at 64.4°C and 1'30'' at 72°C; and a final extension of 5' at 72°C. All amplifications were size-verified on agarose gels. Transformations were done as described above. Cells were then plated on solid YPD medium with nourseothricin (100 mg/L) for *MATa* strains transformed with *DHFR[1,2]-NatMX4* cassettes or hygromycin B (250 mg/L) for *MATα* strains transformed with *DHFR[3]-HPH* cassettes. For *Skud*, G418 was added in medium (200 mg/L) because FM1109 and FM1110 strains had the *KanMX4* resistance cassette and this selection eliminates the possibility that the selection marker of the DHFR cassettes would simply recombine with the *KanMX4* cassette. Cells were incubated at least for 3 days at specific temperatures (Table S3). Colonies were isolated and the correct fusion of the *DHFR[1,2]-NatMX4* or *DHFR[3]-HPH* cassette with the targeted gene was controlled for by colony PCR (see above). The forward primer used for PCR amplification was homologous to 20-22 bp of a sequence located 50-100 bp upstream of the 3' end of the targeted gene and was specific to the species and the gene (See Table S5). The reverse primers ADHTerm-R (Table S4) targeted a 20 bp sequence located after the 3' end the *DHFR[1,2]* and the *DHFR[3]* fragments and was common to both cassettes. The amplified fragment thus contained the 50-100 bp end of the coding sequence of the gene, the linker and the *DHFR[1,2]* or the *DHFR[3]* fragment. PCR products were size-verified on gels. If they were of appropriate length, they were systematically sequenced by standard Sanger sequencing with the ADHTerm-R primer, to ensure that insertions, deletions or non-synonymous mutations did not occur during the construction. Around 20% of the positive colonies were invalidated at this stage mostly because of small deletions and non-synonymous mutations in the fusion area (including the 3’ end of the targeted gene and the linker). Strains from the *Scer* DHFR collection also passed the same quality controls [[2](#_ENREF_2)]. Strains with an incorrect sequence were systematically discarded. Eight strains out of 48 (seven *MATα* and one *MATa*) were not obtained in *Skud* after five attempts (Table S1). On average, *MATα* strains often required more attempts per than *MATa* strains (1.7 and 1.3 respectively). *Suva* strains MG032 and JBL033 Nup145, Nup120 and Nup85 fused to the DHFR fragments were constructed following to the same protocol.

**5. DHFR-PCA screen for PPIs in *Scer*, *Skud* and their hybrids in the NPC and RNApII complexes**. *MATa* and *MATα* strains were combined from fresh liquid cultures onto a 384-position plate of solid YPD, using a Freedom EVO® 150 TECAN© robot-handled pin-tool containing 384 floating metallic pins FPN1 V&P Scientific©, allowing the transfer of the same amount of cells for each strain (Figure S8). Plates were incubated for four days at 25°C. Diploid selection was performed two successive times on a 1536-array on solid YPD with both hygromycin B and nourseothricin. Plates were incubated for two days at 25°C. Diploid cells were then transferred onto solid synthetic medium with MTX (200 mg/L). Plates were incubated for five days at 30°C (Figure S8).

**6. PCA assay image acquisition and analysis**. Pictures of plates were acquired using a 10.1 megapixels camera (Canon EOS RebelXS) (exposition time: 1s; aperture: F5.6; Iso: 100; mode: monochromatic; automatic correction of whites; polarizing filter). Colony size was measured as integrated pixel intensity with a macro implemented in the software ImageJ 1.45m (<http://rsbweb.nih.gov/ij/>). Each picture was converted to binary with a manually determined threshold that was applied to all the plates. A circular selection was then defined at each of the 1536 positions of the array. In these selected areas, particles were detected using the built-in function “Analyze particle” in imageJ, excluding particles touching the edge of the selection and those that had circularity inferior to 0.5 and an area inferior to 20 pixels. In cases where more than one particle was detected, only the closest from the selection center was selected. This particle was considered as a colony only if its mass center was not more distant from the selection center than half the distance between two positions. In the same way, when only one particle was detected, it was validated as a colony according to the same criteria. All plate images were also manually examined. Integrated density of each colony was measured on the original picture. In each selection area, integrated density of the background (i.e. pixels below the threshold in the binary picture) was also measured. This background was then subtracted from the colony intensity to obtain the final colony intensity value. On each MTX plate, colony size values were corrected by the median value of each plate to allow comparisons, and then log_10_ transformed. Colonies corresponding to empty positions at the second round of selection on YPD with antibiotics were systematically removed from the analyses. Analyses were then carried out from mean index value calculated among triplicates of each pairwise combination in each species and each hybrid. This mean growth signal index (SI) was used in following statistical analysis.

**7. Replacement of *ScerNUP145* by *SkudNUP145* in a haploid strain.** In order to replace the *Scer NUP145* (*ScerNUP145*) coding sequence with its homolog sequence from *Skud* (*Skud NUP145*), *SkudNUP145* gene was amplified in a first PCR reaction from *Skud* ZP591 genomic DNA (primers OP37-A1 and OP37-C3, see Table S4). A second PCR reaction was used to amplify ADH terminator and *NATMX* marker from pAG25-L-DHFR[1,2] plasmid (primers OP37-D3 and OP37-B1, see Table S4). The two PCR reactions were performed using KAPA HiFi™ HotStart DNA polymerase (Kapa Biosystems) as described above. Transformations were performed in *Scer* BY4741 and selected on YPD/nourseothricin as described above. Co-transformation of BY4741 with the two PCR products was possible because a 40 nt homology region was present at the junction between the two PCR products. The *Scer* strain expressing *SkudNUP145* was confirmed by PCR product sequencing to ensure that homologous recombination took place at the expected site and that there was no partial recombination between *Scer NUP145* and *Skud NUP145* generating a chimeric sequence. Three *NUP145* fragments covering the whole gene were amplified and sequenced by combining respectively primers OP37-E01 with OP37-H01, OP32-E08 with OP37-E02 and OP37-D02 with ADHTerm-R (See Table S4).

**References**

1. Güldener U, Heinisch J, Köhler G, Voss D, Hegemann J (2002) A second set of loxP marker cassettes for Cre-mediated multiple gene knockouts in budding yeast. Nucleic Acids Res 30: e23.

2. Tarassov K, Messier V, Landry CR, Radinovic S, Serna Molina MM, et al. (2008) An in vivo map of the yeast protein interactome. Science 320: 1465-1470.

3. Huxley C, Green ED, Dunham I (1990) Rapid assessment of S. cerevisiae mating type by PCR. Trends Genet 6: 236.

4. Steensma HY, Ter Linde JJ (2001) Plasmids with the Cre-recombinase and the dominant nat marker, suitable for use in prototrophic strains of Saccharomyces cerevisiae and Kluyveromyces lactis. Yeast 18: 469-472.

5. Amberg DC, Burke D, Strathern JN, Cold Spring Harbor Laboratory. (2005) Methods in yeast genetics : a Cold Spring Harbor Laboratory course manual. Cold Spring Harbor, N.Y.: Cold Spring Harbor Laboratory Press. xvii, 230 p. p.

6. Manderson EN, Malleshaiah M, Michnick SW (2008) A novel genetic screen implicates Elm1 in the inactivation of the yeast transcription factor SBF. PLoS One 3: e1500.

7. Malleshaiah MK, Shahrezaei V, Swain PS, Michnick SW (2010) The scaffold protein Ste5 directly controls a switch-like mating decision in yeast. Nature 465: 101-105.

8. Michnick SW, Ear PH, Landry C, Malleshaiah MK, Messier V (2010) A toolkit of protein-fragment complementation assays for studying and dissecting large-scale and dynamic protein-protein interactions in living cells. Methods in Enzymology 470: 335-368.

9. Scannell DR, Zill OA, Rokas A, Payen C, Dunham MJ, et al. (2011) The Awesome Power of Yeast Evolutionary Genetics: New Genome Sequences and Strain Resources for the *Saccharomyces sensu stricto* Genus. Genes Genomes Genetics 1: 11-25.
