# Supplementary material for: Evidence for the Robustness of Protein Complexes to Inter-Species Hybridization
Source: PLoS Genet. 2012 Dec 27;8(12):e1003161. doi: 10.1371/journal.pgen.1003161 (PMC3531474; doi:10.1371/journal.pgen.1003161)
Supplement: Figure S8 — Comparison of SI values after correction by regression analysis between Scer and hybrid 2 (left) or hybrid 1 (right). (A) Estimation of the SI threshold value (t) for the detection of PPIs in Scer. (B) Estimation of the SI threshold value (t) for the detection of PPIs in hybrids. In each figure, distribution of SI values (log10) is showed with solid lines representing SI values measured within complexes (SW values) and dotted lines representing SI values measured among complexes (SA values). The t value corresponds to the maximal SA value measured in the diploid. Grey frames indicate background growth (SI<t). (C) Overlapped networks of Scer and hybrids Only SI above t and comparable interactions are represented. Line width is proportional to SI values measured between proteins in Scer (red) and hybrids (green) in the NPC (left) and the RNApII (right). Interactions appear in brown when Scer and hybrid SI values overlap. Different degrees of brown depend on whether the interaction could be tested in reciprocal ways or not. (PDF) [file pgen.1003161.s010.pdf]

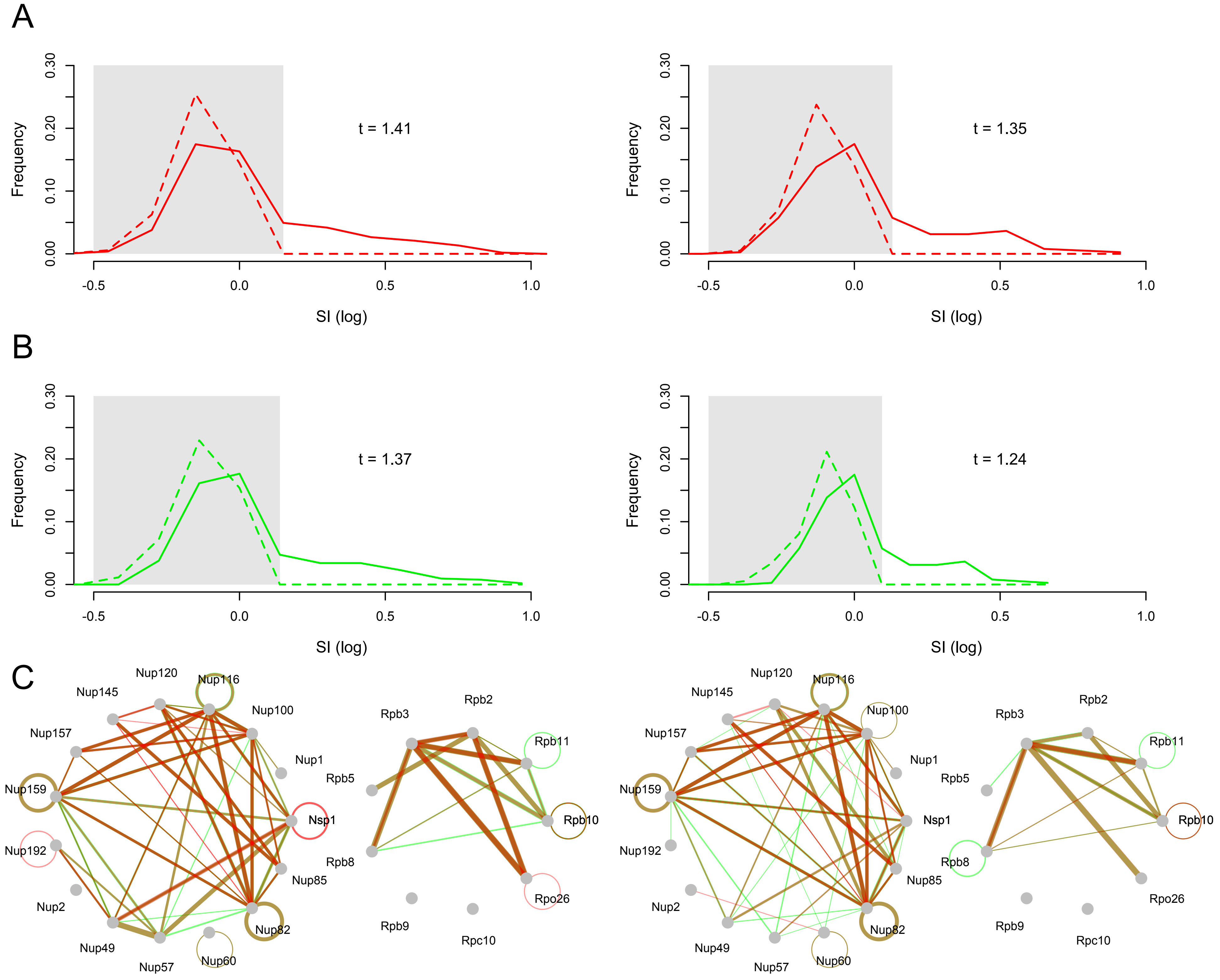

A
Frequency
SI (log)
t = 1.41
t = 1.35
B
t = 1.37
t = 1.24
C
Nup120
Nup116
Nup145
Nup100
Nup157
Nup1
Nup159
Nsp1
Nup192
Nup85
Nup2
Nup82
Nup49
Nup57
Nup60
Rpb3
Rpb2
Rpb11
Rpb5
Rpb10
Rpb8
Rpo26
Rpb9
Rpc10
